# Electricity system based on 100% renewable energy for India and SAARC

**DOI:** 10.1371/journal.pone.0180611

**Published:** 2017-07-19

**Authors:** Ashish Gulagi, Piyush Choudhary, Dmitrii Bogdanov, Christian Breyer

**Affiliations:** 1 Lappeenranta University of Technology, Lappeenranta, Finland; 2 Indian Institute of Technology (BHU), Varanasi, India; University of Liverpool, UNITED KINGDOM

## Abstract

The developing region of SAARC (South Asian Association for Regional Cooperation) is home to a large number of people living below the poverty line. In future, providing affordable, universally accessible, reliable, low to zero carbon electricity in this region will be the main aim. A cost optimal 100% renewable energy system is simulated for SAARC for the year 2030 on an hourly resolved basis. The region was divided into 16 sub-regions and three different scenarios were set up based on the level of high voltage direct current (HVDC) grid connections. The results obtained for a total system levelised cost of electricity (LCOE) showed a decrease from 71.6 €/MWh in a decentralized to 67.2 €/MWh for a centralized grid connected scenario. An additional scenario was simulated to show the benefits of integrating industrial gas production and seawater reverse osmosis desalination demand, and showed the system cost decreased by 5% and total electricity generation decreased by 1%. The results show that a 100% renewable energy system could be a reality in the SAARC region with the cost assumptions used in this research and it may be more cost competitive than nuclear and fossil carbon capture and storage (CCS) alternatives. One of the limitations of this study is the cost of land for installation of renewables which is not included in the LCOE calculations, but regarded as a minor contribution.

## 1. Introduction

Energy is critical, directly or indirectly, to the entire process of evolution, growth and survival of all living beings. In addition, it plays a vital role in the socio-economic development and human welfare of a country, and any uncertainty in its supply can threaten the functioning of an economy, particularly in developing countries [1]. The region of interest for this research is the developing region of South Asia, which is made up of the following countries: Afghanistan, Bangladesh, Bhutan, India, Maldives, Nepal, Pakistan, and Sri Lanka. Collectively, they are also called SAARC (South Asian Association for Regional Cooperation). Providing affordable, universally accessible, reliable, low to zero carbon electricity in the developing countries will be the main aim of electricity generation in the next decades [2]. A report published by WWF lists ten recommendations for a 100% renewable energy (RE) future. The top two recommendations include, firstly, developing new and existing renewable energy sources to provide clean energy, and secondly, exchange of clean energy through grids, making use of sustainable resources in different areas [3]. A least cost energy system needs to be obtained without compromising the above mentioned objectives.

There is a need for sustainable energy supply as 87% of global (and SAARC region) energy supply is not sustainable [4]. The need for a sustainable energy system is eminent due to lack of availability of non-renewable resources, environmental consequences [5], or severe lasting security problems for nuclear power [6]. The SAARC region has a vast potential for sustainable energy due to the availability of abundant solar energy, vast land mass, sea waves, bioenergy, rivers, windy areas, mountains and other natural means. Harvesting the vast, available renewable energy resources is the way forward in achieving sustainable development.

The largest country in the SAARC region in terms of population and gross domestic product (GDP) is India with 1.2 billion inhabitants which accounts for 17% of world population [7]. India is the fourth-largest consumer of energy, accounting for 4.9% of global consumption, which is dominated by coal and imported oil [8]. With a high rate of population growth and GDP expected to grow at 8% per annum till 2030 and 6% beyond that [9], it is evident that the demand for electricity is expected to grow in the future [10]. As a matter of fact, in 2014, 240 million people in India did not have access to electricity, while 840 million people relied on wood, crop waste, dung and biomass to cook in traditional cook stoves, which is the major cause of indoor air pollution and premature death [11]. Climate change will affect most Indians due to flooding, change in the monsoon cycle and water scarcity [12, 13].

Coal is India’s primary source of energy and the country is the world’s third largest coal producer after China and the United States [14]. Coal is followed by oil and gas generation. According to International Monetary Fund, India has huge subsidies for coal and other fossil fuels [15]. India’s post tax subsidies for coal for the year 2015 were at 196 bUSD. Also, the government supports coal mining through research and development and several tax benefits for coal transportation [16]. Further, coal-fired power plants are associated with high health costs and heavy metal emissions [17, 18, 19] which are actually not yet taken into account in India in optimizing the societal cost of energy supply. The total subsidies for coal-based and gas-based electricity generation can be estimated for the year 2010 at 84 €/MWh_el_ and 15 €/MWh_el_, based on estimated total subsidies of 8.7 USD/GJ (coal) and 2.2 USD/GJ (gas) according to the IMF [20], primary energy demand for electricity of 2338 TWh_th_ (coal) and 302 TWh_th_ (gas), electricity generation of 653 TWh_el_ (coal) and 118 TWh_el_ (gas) according to the IEA [21] and long-term USD/€ exchange rate of 1.3. Taking away the subsidies from the fossil fuels and investing in RE would enable India to achieve the climate change mitigation goals and zero carbon emissions.

The annual Conference of Parties (COP) 21 held in Paris during December 2015 was an action driven event, when the fight against climate change took a dramatic turn. The conference presented political and business leaders with the opportunity to take the critical decisions needed to keep average temperature rise to no more than 1.5 or 2 degrees Celsius, which finally requires net zero greenhouse gas emissions shortly after the middle of this century [22]. Deliberations among country representatives observed that dynamic change is happening in energy supply, but important is that change needs to happen faster. The Energy [R]evolution scenario of Greenpeace in cooperation with the German Aerospace Center [23, 24] proposes a pathway to restrict global CO_2_ emissions and in turn restricting temperature rise to 2°C by including renewable energy sources and phasing out nuclear energy.

The International Energy Agency [11] has projected India at the centre of the world energy stage in terms of projected rise in energy demand and will contribute the single largest share of around one-quarter in global energy demand by the year 2040. With policies in place to accelerate the country’s modernization and develop its manufacturing base (via the “Make in India” programme), population and incomes on the rise and an additional 315 million people anticipated to live in India’s cities by 2040, India is entering a sustained period of rapid growth in energy consumption [11].

But the good news for India is the effort and the will shown by the government to provide electricity for all in a sustainable way while reducing the effects of climate change. According to Shearer et al.[25], the average cost of electricity produced from coal power plants in 2020 is more expensive than solar PV and onshore wind and this will lead to underutilization or stranded coal plants. Already there has been decrease in average plant load factor which fell from 79% to 64% from 2007 to 2015 [26]. This has given rise to reduced demand of imported coal and it was evident from the statistics, as coal imports fell by 15% from the last year in April 2016 [27]. According to the energy minister of India, a new coal power plant will give costlier power than a solar plant, which is due to the rapid decrease in solar prices in recent years [28]. This is evident from the government’s plan to scrap 16 GW ultra-mega coal fired power plants [29]. The Central Electricity Authority [30] in its draft National Electricity Plan specifically mentions that till 2022 India does not require any more coal based capacity to be added above the current levels.

India has ambitious plans to expand the deployment of solar and wind power. The targeted levels of deployment for renewables is 175 GW by 2022 (of which 100 GW is solar and 60 GW is wind), a powerful statement of intent from the Government of India [31]. According to the draft electricity plan published by the government of India, it forecasts around 54% of India’s total electricity capacity will come from renewables, thereof 43% new renewables and 11% hydropower, 2% from nuclear energy and 44% from fossil power plants by 2027 and the Paris Agreement target was 40% by 2030 [30]. The Government’s goal of ‘Electricity for All’ to be achieved would require huge investments, infusion of new technology and international support [32]. The main renewable energy driver is ‘The National Solar Mission’, which aims to promote the development and use of solar energy for power generation with an ultimate aim of making solar cost competitive with fossil based energy options through long term policy, large scale deployment goals, aggressive R&D and domestic production of critical raw materials [33]. India offers huge growth potential for the solar PV industry.

During COP 21, India launched the International Solar Alliance (ISA) with countries located in between the Tropic of Cancer and Tropic of Capricorn [34]. ISA is conceived as a coalition of solar resource rich countries to address their special energy needs and will provide a platform to collaborate on addressing the identified gaps through a common, agreed approach. It will not duplicate or replicate the efforts that others (like International Renewable Energy Agency (IRENA), Renewable Energy and Energy Efficiency Partnership (REEEP), International Energy Agency (IEA), Renewable Energy Policy Network for the 21st Century (REN21), United Nations bodies, bilateral organizations etc.) are currently engaged in, but will establish networks and develop synergies with them and supplement their efforts in a sustainable and focused manner.

Human development and economic growth in the SAARC region and particularly in India can be achieved through growth in energy use and its spread to all remote areas. Electricity will play an important role in improving human development and quality of human life. For India, economic growth hinges on bringing the rural population out of the dark and providing clean, continuous electricity supply. Electricity generation from renewable energy sources in a decentralized manner is one of the options to meet rural electricity needs [35, 36]. For locations far away from the existing grid or where grid extension is not possible, economically or technically, decentralized generation would provide basic electricity and likely overcome the problem of frequent blackouts. Long term planning will be essential for energy security, which will be based on renewable resources in a centralized and decentralized manner, and will help in sustainable development as well as minimize the carbon footprint.

For SAARC and India there is no research yet on the sustainable energy transition pathways into the future decades or none of them integrated all aspects in the required manner. The list of various future scenarios for SAARC and India with the key findings is given in Table 1. However, none of them considered the following approaches as applied in this study, such as hourly based model that guarantees that the hourly total electric energy supply in a year in the sub-regions covers the local demand from all sectors (which is most relevant during the monsoon season); different transmission grid development levels that are able to reduce the need of energy storage and total costs; and an integrated scenario that assumes electricity demand, water desalination and industrial gas demand.

**Table 1:** Key findings of different scenario studies for SAARC.

Study	Scope	Key findings
Abhyankar N. and Phadke A., [37]	India	Based on the simulation results of the hourly grid dispatch simulation for the year 2047. Various scenarios were simulated. In the minimum emissions scenario, installed capacities of solar and wind is 930 and 472 GW, respectively.
IEA [11]	India	For the year 2030, the installed capacity of fossil fuels is 419 GW and renewables is 462 GW. Solar PV contributes 100 GW and wind 102 GW
Teske S. et al., [23]	all countries including India	The share of renewables in the electricity generation would be 56% (2030) and 93% (2050). The installed capacities of the renewables will reach 770 GW (2030), 2240 GW (2050) and 100% RE scenario 3260 GW. PV installed capacities of 390 GW and wind 449 GW in 2030.
Powergrid Corporation of India, [38]	India	Deserts from the western part and northern part of India would be utilized to power the electricity demand for the whole country. Mainly powered by solar and wind, which have installed capacity of about 485 GW
TERI and WWF-India [9]	India	In the 100% RE scenario for 2051, installed capacity for solar is 1200 GW, offshore wind 1113 GW and onshore wind 117 GW. The total installed capacity would be 2870 GW.
WWF-India and WISE [36]	For the state of Kerala, India	In a 100% RE scenario for 2050, solar contributes 51% and wind contributes 24% of the total electricity generation mix
Teske S. et al., [39]	India	The share of renewables in the electricity generation would be 32% (2020), 62% (2030) and 92% (2050). Wind, solar thermal energy and PV will contribute 74% of electricity generation. The installed capacities of renewables will reach 548 GW in 2030 and 1356 GW by 2050.
Teske S. et al., [40]	India	The share of renewables in electricity generation would be 69% by 2050, with an installed capacity of 1659 GW.

## 2. Methodology

**Fig 1:**
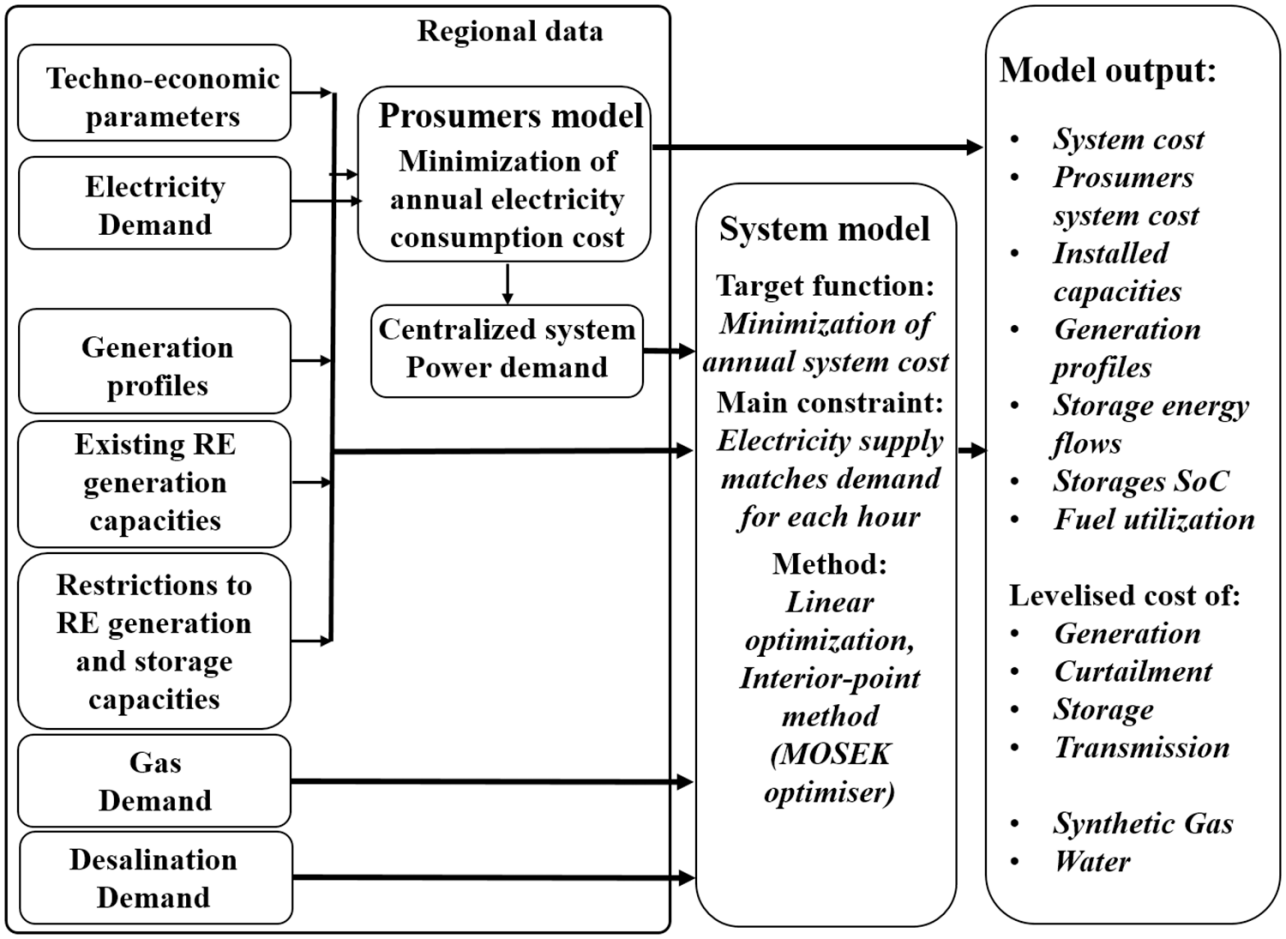
Model flow diagram with the input data, system model optimization and output data.

The model applied to the simulation uses linear optimization for the energy system parameters under previously defined limitations which are applied to the system and the assumptions for the future RE power generation and demand. The detailed description of the model can be found in Bogdanov and Breyer [41], but the main functionalities are summarised in the following sections. Required storage technologies, including additional water desalination and synthetic natural gas generation, are the flexible demands in the model. One key limitation for the system optimization is that demand should be satisfied by power generation on an hourly basis for an entire year as shown in Eq 1. To obtain a least cost energy system is the main target of system optimization. The costs are calculated as sum of the annualised costs of all installed capacities of the different technologies, energy generation and generation ramping. Also, the system consists of PV prosumers for residential, commercial and industrial sectors. The term prosumer is used to refer to energy consumers who also produce their own power from a range of different onsite generators, e.g. diesel generators, combined heat-and-power systems, wind turbines, and PV systems [42]. In this study, only onsite consumption and generation from PV systems are considered and termed as prosumers [43, 44]. The PV prosumers install the required individual capacities of rooftop PV systems and batteries. Minimizing cost of consumed energy is the target function for the prosumers. The cost of consumed energy is calculated as sum of PV self-consumption, annual cost and cost of electricity consumed from the grid. The prosumers can sell electricity to the grid at 2 €ct/kWh, however they have to satisfy their own demand before selling. The flowchart of the model is presented in Fig 1.

$$\forall h\in \left[1,8760\right]\;\left(\sum_{t}^{tech}E_{gen,t}\right),h+\left(\sum_{r}^{reg}E_{imp,r}\right),h+\left(\sum_{t}^{stor}E_{stor,disch}\right),h=\left(E_{demand}\right),h+\left(\sum_{r}^{reg}E_{exp,r}\right),h+\left(\sum_{t}^{stor}E_{stor,ch}\right),h+\left(E_{curt}\right),h$$ (1)

$$min\;\left(\sum_{r=1}^{reg}\sum_{t=1}^{tech}\left(CAPEX_{t}\cdot crf_{t}+OPEXfix_{t}\right)\cdot instCap_{t,r}+OPEXvar_{t}\cdot E_{gen,t,r}+rampCost_{t}\cdot totRamp_{t,r}\right)$$ (2)

The main constraint of the system optimization is given in Eq 1. It is defined as for every hour of a year in a particular region, electricity generation from all the technologies (*E*_*gen*,*t*_), imported electricity from the regions (*E*_*imp*,*r*_*)* and electricity from storage discharge (*E*_*stor*,*disch)*_ should be equal to the total demand for an hour (*E*_*demand*_), electricity exported to other regions (*E*_*exp*,*r*_), electricity for charging storage technologies (*E*_*stor*,*ch*_)and curtailed electricity (*E*_*curt*_). The other abbreviations used in this equation are: hours (*h*), technology (*t*), all technologies used in modelling (*tech*), sub-region (*r*), all sub-regions (*reg*). Eq 2 provides the target function for system optimization. The abbreviations used here include (*CAPEX*_*t*_)—capital cost of each technology, (*crf*_*t*_)–capital recovery factor for each technology, (*OPEXfix*_*t*_)–fixed operational cost for each technology, (*OPEXvar*_*t*_)—variable operational cost each technology, installed capacity in a region (*instCap*_*t*,*r*_), electricity generation by each technology (*E*_*gen*,*t*,*r*_), ramping cost of each technology (*rampCost*_*t*_) and annual total power ramping values for each technology (*totRamp*_*t*,*r*_). The balancing of the system is mainly done by gas peakers, fuelled by biomethane or synthetic natural gas based on power-to-gas technology. The open cycle gas turbine (OCGT) and combined cycle gas turbine (CCGT) power plants can be ramped within a few minutes and 60 minutes, respectively[45, 46], which is within the resolution of the applied model. Base generation plants do not exist in the model, except hydro run-of-river, however there is of course variation due to the hydro resource availability. Batteries can react on millisecond scale and can even receive control information from the frequency in the grid.

The equations used for calculating total levelized cost of electricity (LCOE), primary LCOE, levelized cost of curtailment (LCOC), levelized cost of storage (LCOS), levelized cost of transmission (LCOT), total annual cost of the system and total capital cost are presented below (Eqs 3–9)

$$totalCost\;system=\sum_{r}^{reg}LCOE_{r}\cdot E_{demand,r}$$ (3)

$$LCOE_{r}=LCOE_{prim,r}+LCOC_{r}+LCOS_{r}+LCOT_{r}$$ (4)

$$LCOE_{prim_{r}}=\;\frac{\sum_{t=1}^{REtech}\left(CAPEX_{t}\cdot crf_{t}+OPEXfix_{t}\right)\cdot Cap_{t,r}+OPEXvar_{t}\cdot E_{gen,t,r}}{E_{demand,r}+E_{exp,r}-E_{imp,r}}$$ (5)

$$LCOC_{r}=\;LCOE_{prim}{}_{{}_{r}}\cdot \;\frac{E_{curt,r}}{E_{demand,r}+E_{exp,r}-E_{imp,r}}$$ (6)

$$LCOS_{r}=\;\;\frac{\sum_{t=1}^{Storagetech}\left(CAPEXcrf_{t}+OPEXfix_{t}\right)\cdot Cap_{t,r}+OPEXvar_{t}\cdot E_{storage,disch,t,r}}{E_{demand,r}+E_{exp,r}-E_{imp,r}}$$ (7)

$$LCOT_{r}=\;\frac{totalCost_{TR}\cdot share_{r}}{E_{demand,r}+E_{exp,r}-E_{imp,r}}$$ (8)

$$CAPEX_{tot}=\sum_{r}^{reg}\sum_{t}^{tech}CAPEX_{t}\cdot Cap_{t,r}$$ (9)

### 2.1 Input data for the model

Detailed information of the input data used for the model is given in Bogdanov and Breyer [41] and additional calculations related to geothermal energy, desalination water demand and industrial gas demand data are described here.

- The potential for geothermal energy for the sub-regions is calculated on the available information related to heat flow rate and ambient temperature of the surface [47, 48] for the year 2005. For the sub-regions where the heat flow data were not available, extrapolation was performed to get the required data. Based on the available data, different temperature levels and available heat at the mid-point of a 1 km thick deep layer and for between the depths of 1 km to 10 km [49, 50, 51] globally with 0.45° x 0.45° spatial resolution, the required potential is derived.
- For every sub-region, projected water desalination demand is calculated as projections for water consumption and stress level [52] with an assumption that water stress more than 50% will be covered by desalinated seawater. Detailed calculations for the technical constraints and financial cost of seawater reverse osmosis desalination are described Caldera et.al. [53].
- Natural gas consumption was derived in the non-energy sector for every sub-region. Industrial gas consumption data are based on IEA statistics for non-energy sector demand [54].

### 2.2 Applied technologies

For the SAARC region, technologies used for energy system optimization can be divided into four main categories

#### • Technologies for converting renewable energy sources into electricity

The technologies used for transforming RE sources into electricity are: two different types of ground mounted PV systems (optimally fixed tilted and single-axis north-south oriented horizontal continuous tracking), rooftop PV for prosumers, concentrating solar thermal power (CSP), onshore wind turbines, hydro power divided into run-of-river and dams, biomass which is divided into biogas and solid biomass, waste-to-energy and geothermal power plants.

#### • Energy storage

The energy storage technologies utilized in the model are system and prosumer batteries, pumped hydro storage (PHS), adiabatic compressed air energy storage (A-CAES), thermal energy storage (TES) and power-to-gas (PtG) technology. Technologies such as water electrolysis, methanation, CO_2_ scrubbing from air, gas storage, and both combined and open cycle gas turbines (CCGT, OCGT) are part of the synthesis of synthetic natural gas (SNG) and its reconversion to electricity. The PtG technologies have to be operated in synchronization because of the absence of hydrogen and CO_2_ storage. As part of the system also there is a biogas buffer storage for 48 hours and part of the biogas can be upgraded to biomethane and introduced to the gas storage.

#### • Energy bridging technologies

The bridging technologies used in this model provide the required flexibility to the energy system in terms of reducing the overall cost of an optimized system. For example, gas produced from PtG can be used for industrial gas demand rather than storage for the electricity sector. Similarly, for producing clean water, excess electricity is utilized by seawater reverse osmosis (SWRO) coupling water and electricity sectors.

#### • Electricity transmission technologies

Electricity transmission inside the sub-regions is assumed to be based on alternating current (AC) grids and not included in the model, and between the sub-regions on high voltage direct current (HVDC). Loss of electricity is due to the transmission lines and in converter stations at the interconnection with the AC grid.

**Fig 2:**
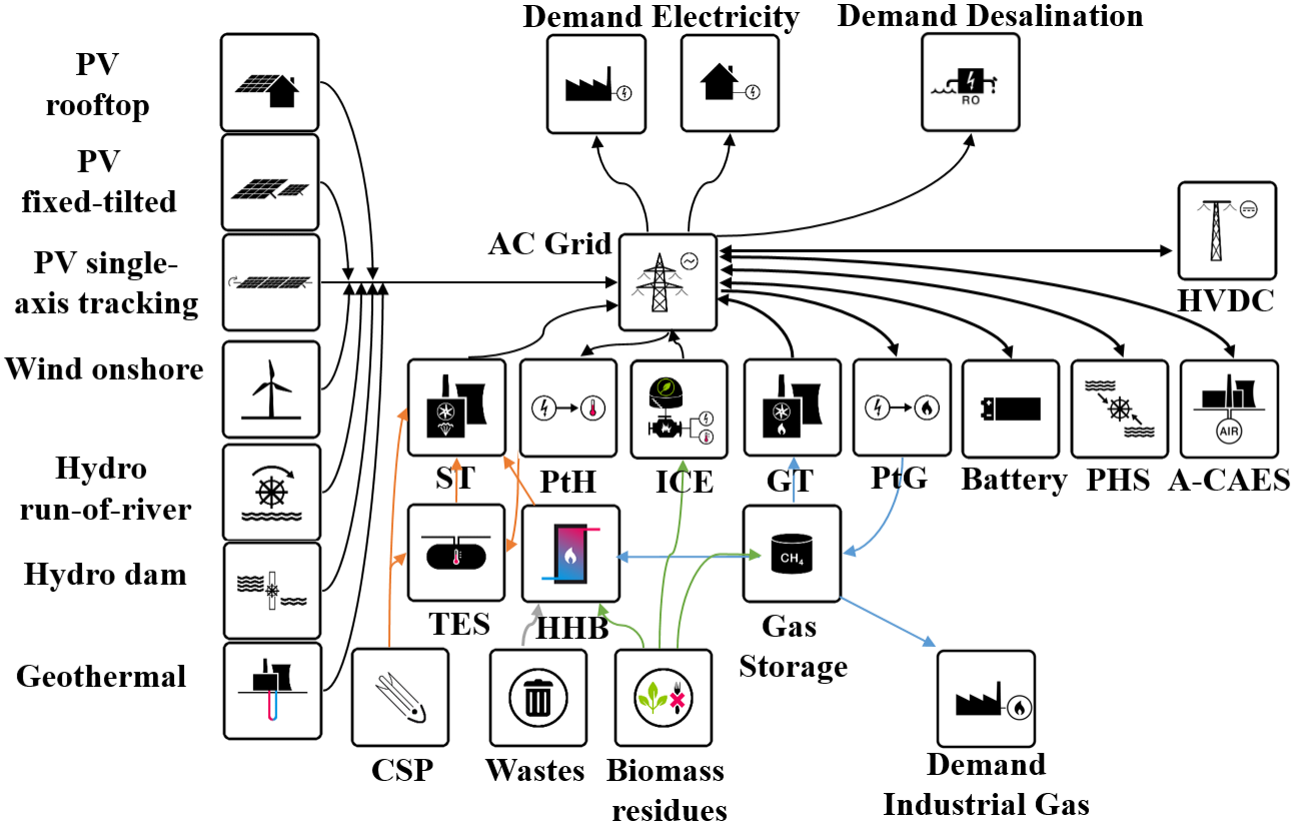
Block diagram of the all the energy technologies applied in the model for the SAARC region.

The full block model diagram is presented in Fig 2.

## 3. Scenario assumptions for the SAARC region

### 3.1 Subdivision of the region and grid structure

**Fig 3:**
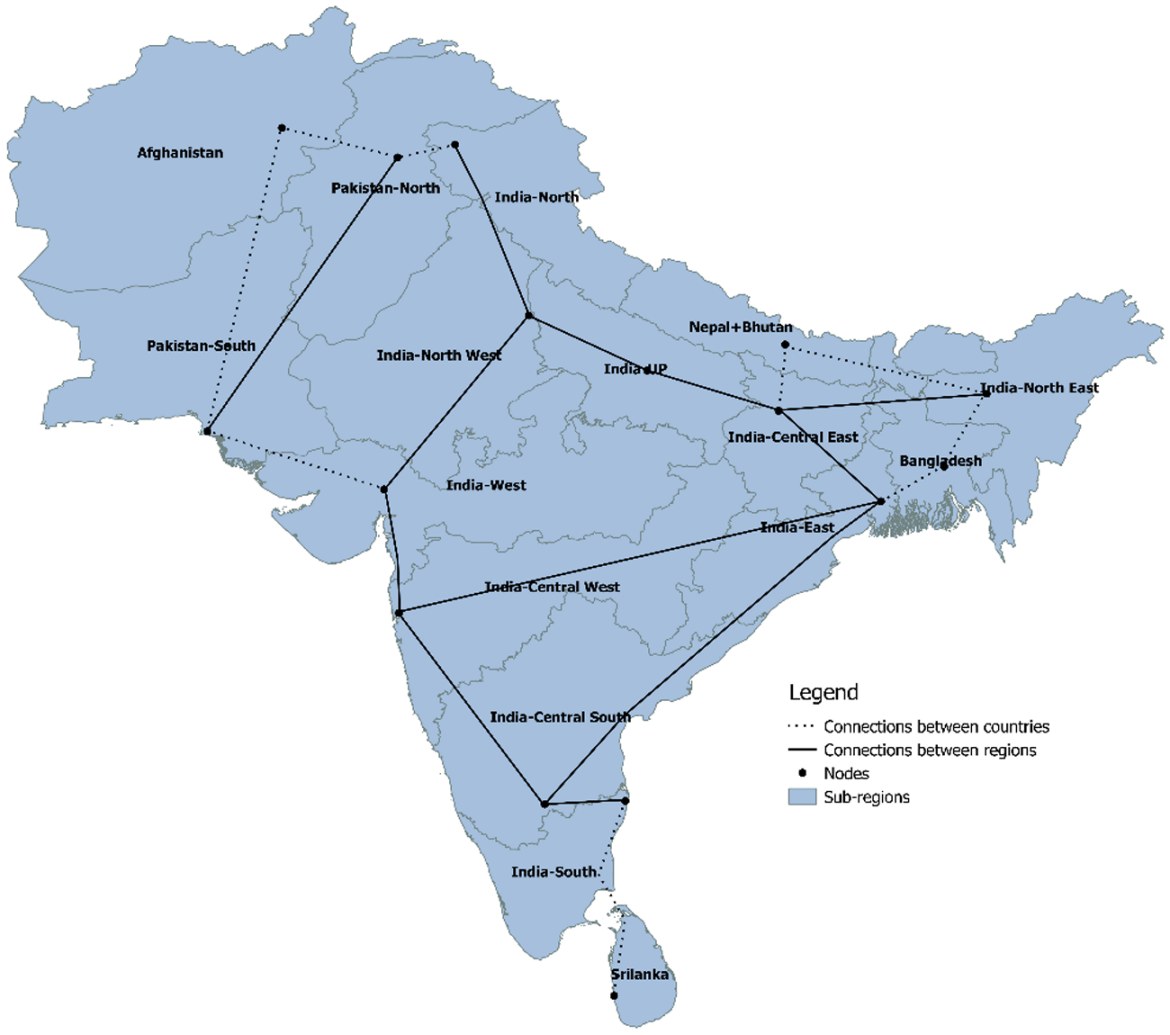
The different SAARC regions and HVDC grid configuration.

The SAARC region is subdivided into 16 sub-regions, according to population distribution, electricity consumption and countries’ grid structure. The different sub-regions can be seen from Fig 3. India is subdivided into 10 different regions, Pakistan into two regions and the other remaining countries are treated as individual regions. The grid connection between the regions is shown in Fig 3, which includes interconnections within the countries shown by dark line and between the countries shown by dotted lines.

### 3.2 Applied scenarios

The different scenarios taken into consideration in this paper for the analysis of the energy system of SAARC region are:

- Region-wide scenario: the regions do not depend on each other and have no interconnections so the demand for electricity is covered by region’s own generation capacity.
- Country-wide scenario: regions of the same country are interconnected via HVDC lines
- Area-wide scenario: energy systems of the countries are interconnected
- Integrated scenario: area-wide scenario plus SWRO desalination and industrial gas demand to provide flexibility to the system where PtG technology also covers industrial gas demand.

### 3.3 Financial and technical assumptions

The model optimization is based on an assumed cost structure and state of technology for the year 2030. The financial assumptions for all the energy system technologies, HVDC lines and converter stations, which are given as net transmission capacity (NTC) for 2030 reference year, are tabulated in Table A of S1 File. The weighted average cost of capital (WACC) is set to 7% (real) for all investments, expect for residential PV prosumers, for which a real WACC of 4% is applied, due to lower financial return requirements. The WACC may not reflect the financing situation in each country but an uniform assumption is required for comparing the results. However, the authors assume that a 7% real WACC will be achieved in the countries of these region by the year 2030. The technical assumptions for energy to power ratios of storage technologies, efficiency numbers for generation and storage technologies and power losses in HVDC transmission lines [55] and converters are presented in Tables B, C and D of S1 File. Price of electricity for residential, commercial and industrial consumers for all the countries is taken from Gerlach et al. [56] and only applied for deriving beneficial self-consumption of PV prosumers. The electricity prices for Nepal and Bhutan are assumed to be similar to India. The electricity prices for 2030 are calculated according to the assumptions that grid electricity prices rise by 5% per annum for <0.15 €/kWh, by 3% per annum for 0.15–0.30 €/kWh and by 1% per annum for >0.30 €/kWh [57]. The electricity prices for all the regions are provided in Table E of S1 File. It should be noted that electricity prices only affect the prosumers of electricity in the model. The renewable energy investments’ financial incentives, such as Renewable Energy Certificate mechanism and Perform Achieve Trade (PAT), are not considered in the assumptions, since a cost-based model is applied.

Table 2 presents the financial assumptions for the storage components utilized in the modelling. For the case of the SAARC region, the cost of batteries will be important as this is the most important storage technology. The general consensus is that, cost will fall as production volumes increase and this is supported by historical cost developments of Lithium-ion batteries. An average value has been used for the battery capex comparing various sources [58, 59, 60, 61, 62, 63].

**Table 2:** Financial assumptions for storage components for year 2030 conditions.

	Capex	Opex fix	Opex var	Lifetime
	[€/kWh]	[€/kWh]	[€/kWh]	[a]
Battery	150	10	0.0002	10
PHS	70	11	0.0002	50
A-CAES	31	0.4	0.0012	40
Thermal energy storage (TES)	24	2	0	20
Gas storage	0.05	0.001	0	50

### 3.4 Feed-in for solar and wind energy

**Fig 4:**
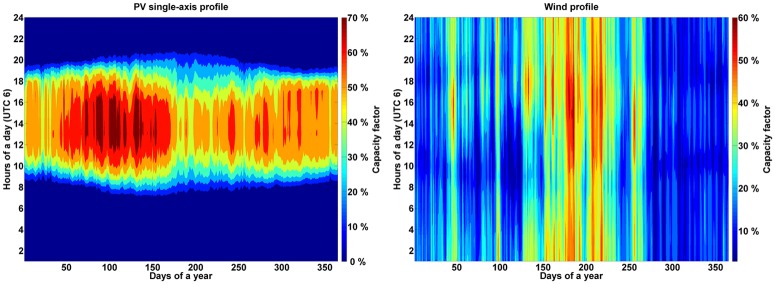
Yearly profile for PV single-axis tracking (left) and wind onshore (right).

Solar CSP, optimally tilted and single-axis tracking PV, and wind energy generation profiles were calculated according to Bogdanov and Breyer [41]. Fig 4 represents aggregated profiles of solar PV generation (optimally tilted and single-axis tracking), wind energy power generation and CSP solar field, normalized to maximum capacity averaged for the SAARC region, are presented in Fig 4. The computed average full load hours (FLH) for optimally tilted, single-axis tracking PV systems, wind power plants and CSP are provided in Table F of S1 File.

For hydro power, generation profiles are computed based on the monthly resolved precipitation data for the year 2005 as a normalized sum of precipitation throughout the regions.

### 3.5 Biomass and geothermal potential

The biomass and waste resources are divided into three categories: Solid wastes, solid residues and biogas. The potential for these resources are taken from [64] and cost associated with all the biomass resources is calculated according to the data from International Energy Agency [65] and Intergovernmental Panel on Climate Change [66]. For solid fuels a 75 €/ton fee for the waste incineration is assumed and it is reflected in the negative cost for solid fuels. The heating values are based on lower heating values (LHV).

The geothermal heat potential for all the regions were calculated based on the spatial data for available heat, temperature and geothermal plants for depths from 1 km to 10 km. For each 0.45° x 0.45° area and depth, LCOE for geothermal is calculated and optimal depth is determined. The assumption for available geothermal heat is that only 10% of it will be utilized as an upper resource limit. The total available heat for all the regions was calculated using the same weighted average formula as for solar and wind feed-in as described in Bogdanov and Breyer [41], with an exception of areas with geothermal LCOE exceeding 100 €/MWh, which are excluded. The calculated potentials for solid biomass, biogas, solid waste and respective costs, and geothermal heat potentials are provided in Tables G and H of S1 File.

### 3.6 Upper and lower limitations on installed capacities

The data for current installed capacities for optimally fixed-tilted PV, wind turbines, hydro power and pumped hydro storage are taken from Farfan and Breyer [67] and summarized in Table I of S1 File. The upper limits for all the above mentioned RE technologies were calculated according to Bogdanov and Breyer [41] and are summarized in Table J of S1 File. It is assumed for biomass residues, biogas and waste to energy plants, that the available and specified amount of the fuel is utilized during the year due to energy efficiency.

### 3.7 Load

**Fig 5:**
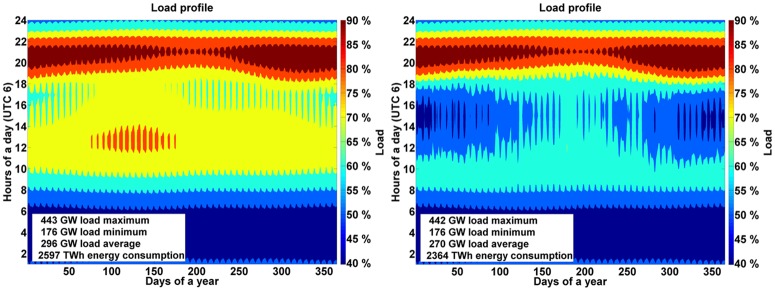
Aggregated load curve (left) and load curve with prosumers influence (right) for the SAARC region for the year 2030.

The load profile for each region is calculated as a fraction of the total demand in that particular country based on synthetic load data weighted by the region’s population. Fig 5 represents the area aggregated demand profile for all the regions considered in SAARC. The additional impact of solar PV prosumers can be observed on the residual load in reduction of overall energy demand and maximum load, by 9% and 0.2%, respectively (Fig 5). The demand for gas in industries and desalination water demand for the SAARC region is given in Table K of S1 File.

## 4. Results

### 4.1 Structure and cost of an optimized energy system

The cost structure of the different scenarios were analysed with the set of parameters calculated as given in Bogdanov and Breyer [41] plus the model extension described in section 2.1. The key financial results for the different scenarios for the SAARC region is presented in Table 3. The results are given as the total system (LCOE) levelised cost of electricity (including PV self-consumption and the centralized system), levelised cost of electricity for primary generation (LCOE primary), levelised cost of curtailment (LCOC), levelised cost of storage technologies (LCOS), levelised cost of transmission (LCOT), total annualized cost, total capital expenditures and other results, which include the total renewable capacity and total primary generation.

**Table 3:** Financial results for the four scenarios applied for the SAARC region.

2030 Scenarios	Total LCOE	LCOE primary	LCOC	LCOS	LCOT	Total ann. cost	Total CAPEX	RE capa-cities	Gener-ated electri-city
	[€/MWh]	[€/MWh]	[€/MWh]	[€/MWh]	[€/MWh]	[b€]	[b€]	[GW]	[TWh]
Region-wide	71.6	42.3	1.5	27.8	0.0	187	1539	1377	2948
Country-wide	69.6	41.9	1.1	25.5	1.1	181	1468	1294	2865
Area-wide	67.2	41.4	0.7	22.7	2.3	174	1421	1210	2818
Integrated scenario	67.9	40.8	1.4	22.6	3.1	299	2562	2213	4988

The benefit due to interconnection of the sub-regions via HVDC power lines has a positive impact on the LCOE and total annual cost of the system, but this impact is only marginal due to limited grid utilization. LCOE and annual cost of the system are decreased by 6.1% and 6.9%, respectively, from region-wide to area-wide scenarios. Also, grid utilization decreases installed capacities and total electricity generated from RE sources by 12.1% and 4.4%, respectively. The cost of transmitting electricity with HVDC power lines is small in comparison to the cost of storage; therefore, total system cost and LCOE are reduced. The transmission lines decrease the need for storage technologies, since energy shifted in time (storage) can partly be cost effectively substituted by energy shift in the location. Also, cost of curtailment is reduced when electricity is transmitted to other sub-regions as seen in Table 3. The components that make up the LCOE in region-wide, country-wide, area-wide and integrated scenarios are presented in Table L of S1 File.

The installed capacities of all the renewable energy technologies show a decrease with increase in installation of HVDC lines as shown in Table 4. The total installed capacities of PV decrease by 16.7% from region-wide to area-wide scenarios due to efficient use of solar resources available in the region. The installed capacity for wind shows a slight increase in the area-wide scenario due to low solar irradiation in the monsoon months. The installed capacities of PV and wind in the integrated scenario increase due to the additional demand of seawater desalination and industrial gas. In the SAARC region, PV is the least cost RE source followed by wind energy. The share of PV single-axis tracking and PV self-consumption of the total solar PV installed capacity for the area-wide scenario is 82.6% and 15.3%, respectively.

**Table 4:** Installed RE technologies and storage capacities for the four scenarios for SAARC region.

		Region-wide	Country-wide	Area-wide	Integrated scenario
PV self-consumption	[GW]	145	145	145	145
PV optimally tilted	[GW]	21	23	3	3
PV single-axis tracking	[GW]	782	721	640	1131
PV total	[GW]	947	889	789	1280
CSP	[GW]	0	0	0	0
Wind energy	[GW]	242	229	245	694
Biomass power plants	[GW]	64	64	61	65
MSW incinerator	[GW]	3	3	3	3
Biogas power plants	[GW]	21	16	22	14
Geothermal power	[GW]	2	6	8	8
Hydro Run-of-River	[GW]	22	21	21	21
Hydro dams	[GW]	35	35	35	35
Battery PV self-consumption	[GWh]	4	4	4	4
Battery System	[GWh]	1389	1522	1450	1682
Battery total	[GWh]	1393	1526	1454	1686
PHS	[GWh]	44	44	44	44
A-CAES	[GWh]	2416	551	3	3187
Heat storage	[GWh]	0	0	0	0
PtG electrolysers	[GW_el_]	43	30	20	115
CCGT	[GW]	50	43	41	99
OCGT	[GW]	3	2	2	0
Steam Turbine	[GW]	0	0	0	0

In the integrated scenario, additional flexible demand from the desalination and industrial gas sectors leads to an increase in installed capacities of the low cost solar by 38.3% and wind resources by 64.7% with respect to the area-wide scenario. There is a slight increase in the installed capacities of the biomass resources. The capacity of the hydro dams does not change as it provides the system with required flexibility. Despite an upper limit of 50% higher than the current capacity considered for hydro dams and hydro run-of-river plants, installed capacities do not increase in the integrated scenario as PV and wind are least cost technologies in the SAARC region.

The generation curves for electricity can be represented for the whole year divided into 8760 hours and sorted according to the generation minus the load, which is represented by a black line as shown in Figure F of S2 File for the area-wide scenario. All the storage technologies used in the system are charged for about 3500 hours in a year, which is due to higher electricity generation than demand. As solar and wind are highly inflexible generation sources, in these particular hours in the SAARC region there is a high electricity generation. The other flexible generation options such as hydro dams, biomass, biogas and discharge of storage technologies are required to balance the high inflexibility. The inflexible electricity generation options reduce significantly in the other hours of the year as the electricity demand decreases and there is a need for flexible electricity generation options, discharge of storage technologies and utilization of the grid. Curtailment of electricity is for only some hundred hours of the year since for all the other hours the HVDC lines enable the export of the electricity from the best RE producing sub-regions to other sub-regions.

### 4.2 Sub-regional analysis on an optimized energy system

**Fig 6:**
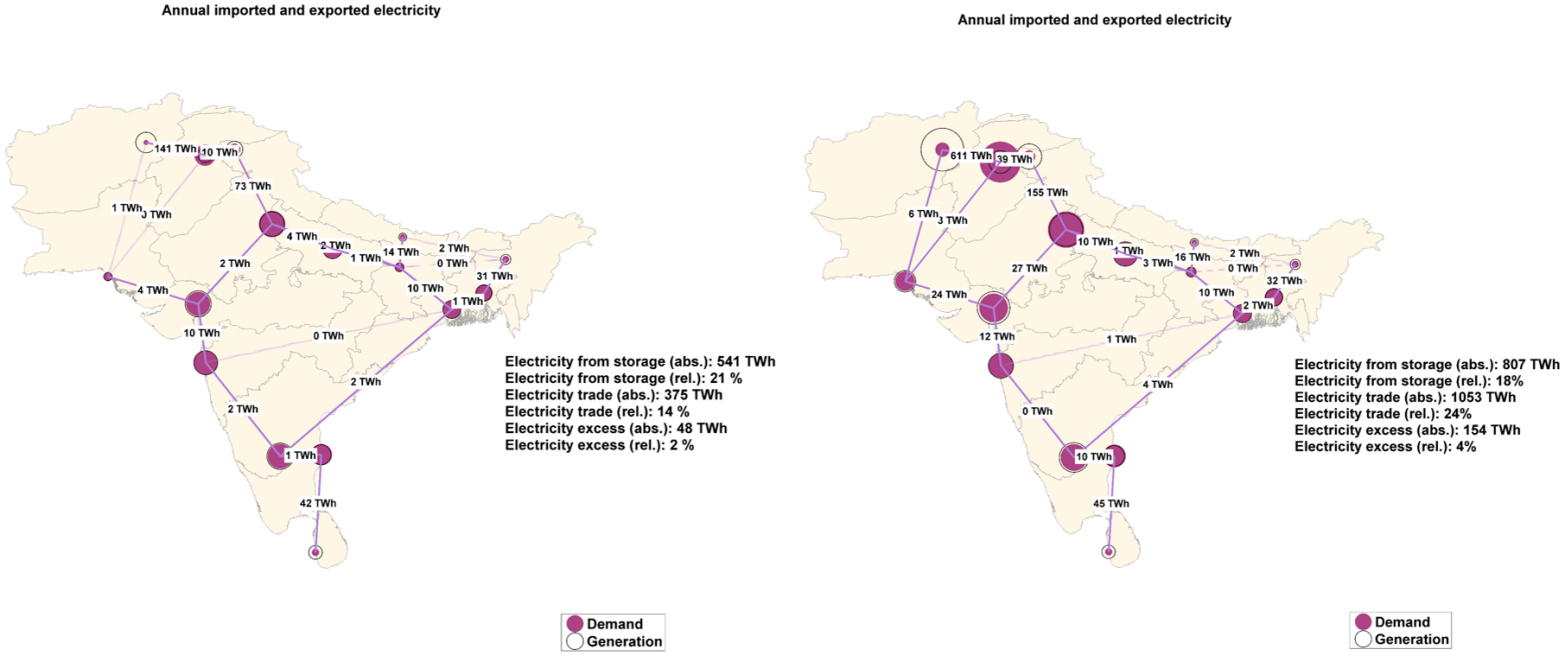
Sub-regional annual import and export of electricity for area-wide (left) and integrated scenario (right).

The sub-regional distribution of system optimized RE resources can be observed from Fig 6. Circles represent demand (solid) and generation (line), with some sub-regions with the best renewable resources represented as net exporters and the others as net importers. The share of export is defined as the ratio of net exported electricity to the generated primary electricity of a sub-region and the share of import is defined as the ratio of imported electricity to the electricity demand. The area average is composed of sub-regional values weighted by the electricity demand. The sub-regions with best renewable energy resources help balance out sub-regions where the availability of renewable resources is scarce. For the region-wide scenario, individual SAARC sub-regions need to match their own demand using their own available RE resources. When the sub-regions are interconnected, as in case of country-wide, area-wide and integrated scenario, sub-regions act as net exporters and importers of electricity. Fig 6 points out the net exporters and importers of electricity in an area-wide and integrated scenario. The differences observed between the demand and generation are mainly due to import and export and also due to losses related to storage. The net exporter regions for SAARC are: Afghanistan, Sri Lanka, India North and India Northeast due to excess of very good RE resources. The net importer regions are: Pakistan North, India Northwest, Bangladesh and India South. Due to a high electricity demand for additional desalination and SNG production, the integrated scenario tends to increase the electricity generation between the regions to fulfil the increased demand. The hourly resolved profiles for Afghanistan, Pakistan North and Sri Lanka are presented in Figures C, D and E of S2 File. The import/export shares in all regions and scenarios are summarized in Table L of S1 File.

The grid utilization profile for the SAARC region can be found in Figure H of S2 File. Electricity trade increases during the night and morning hours almost throughout the year and more so in the monsoon period. This can be explained by the high share of solar PV generation in the region, where electricity is imported by the sub-regions with high inflexible generation. In the monsoon period, due to less solar PV electricity generation, more trading of wind energy takes place within the sub-regions. The capacity of the power lines and grid utilization between the sub-regions for the area-wide open trade scenario is shown in Figure H of S2 File and Table O of S1 File.

**Fig 7:**
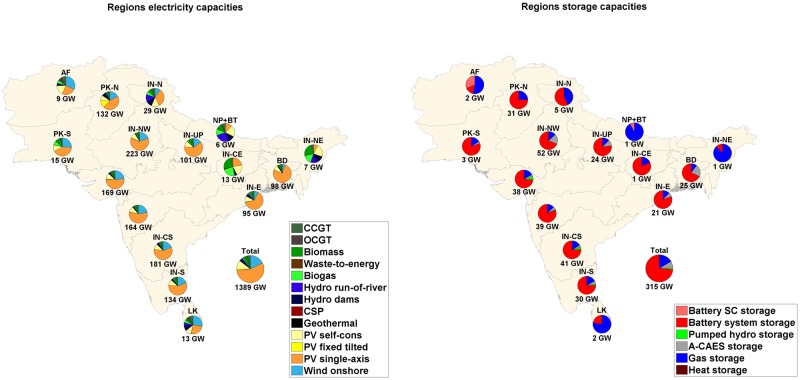
Installed capacities RE generation (left) and storage capacities (right) for the SAARC sub-regions for region-wide scenario.

**Fig 8:**
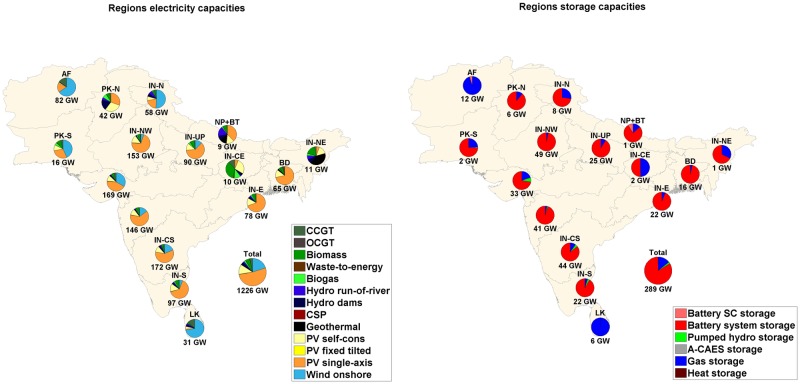
Installed capacities RE generation (left) and storage capacities (right) for the SAARC sub-regions for area-wide scenario.

**Fig 9:**
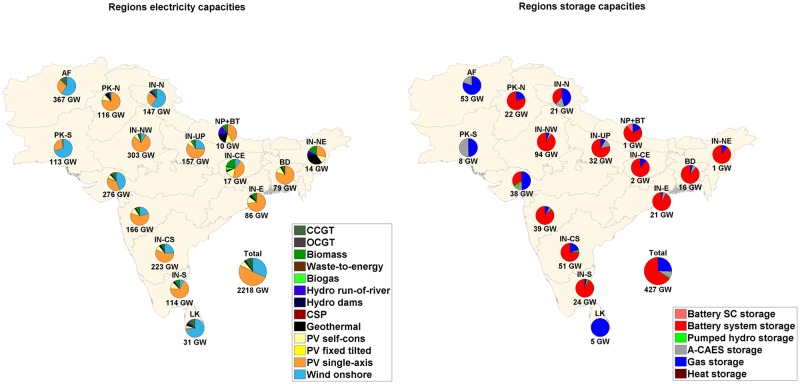
Installed capacities RE generation (left) and storage capacities (right) for the SAARC sub-regions for integrated scenario.

The installed capacities for RE generation and storage technologies for all sub-regions in region-wide, area-wide and integrated scenarios are shown in Figs 7, 8 and 9 respectively. The installed solar PV capacities exceed 50% of the total RE installed capacities in the Western and Southern part of India despite full load hours (FLH) of wind being comparable or exceeding FLH of solar PV. It is observed in the sub-regions that have excellent wind conditions, low cost wind energy is the next preferred technology after solar PV, which is lowest in cost. In Sri Lanka and Afghanistan, installed capacities of wind energy are 71% and 65% of the total RE installed capacity in the area-wide scenario, as these countries have among the best wind resources in the region and export of wind energy takes place from Sri Lanka to high demand centres in Southern India.

The total storage capacity required is greatly influenced by the connection of the sub-regions via HVDC transmission lines, and RE generation and demand in a particular sub-region. Also, the mix of storage technologies for a particular sub-region depends on the above mentioned factors. The throughput of A-CAES, and gas storage technologies decreases by 99.8% and 30.5% respectively, from the region-wide to the area-wide scenario. The discharge capacities, annual throughput of storage technologies and full load cycles per year are provided in Table N of S1 File. State of charge profile diagrams for the area-wide scenario for battery, PHS, A-CAES and gas storage are given in Figure G of S2 File. PV self-consumption does not influence the system in a big way in the SAARC region due to low electricity prices. PV self-generation covers 38.8%, 36.8% and 37.3% of residential, commercial and industrial prosumer demand, respectively. An overview of PV self-consumption is provided in Table M of S1 File.

### 4.3 Energy flow for the optimised power systems for SAARC

**Fig 10:**
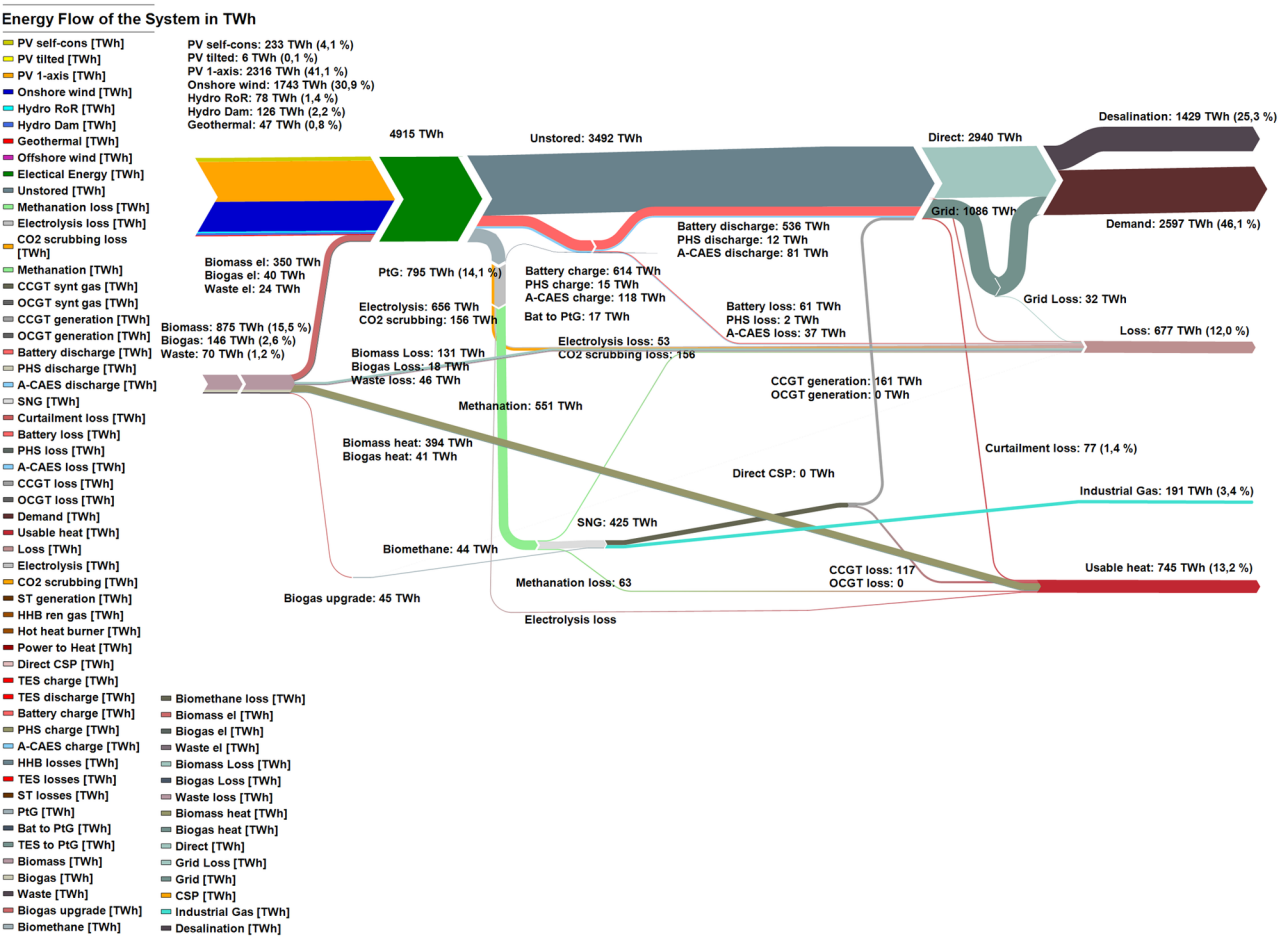
Energy flow of the system in the integrated scenario for the year 2030.

The energy flow of the system from generation to demand for the integrated scenario is presented in Fig 10. The energy flow diagram is made up of RE resources, storage technologies for the generated energy and the transmission of this energy via HVDC grids. The end use of electricity for the integrated scenario consists of electricity, desalination and industrial gas demand. The potentially usable heat generated and the losses incurred are comprised of curtailed electricity, heat produced by biomass, biogas and waste-to-energy power plants, heat generated from electrolysers for transforming power-to-hydrogen, in the methanation process transforming hydrogen-to-methane, and methane-to-power in gas turbines. Efficiency losses incurred in A-CAES, PHS, battery storage and HVDC transmission grid losses form part of the overall losses. The energy flow diagrams for the region-wide and area-wide scenario are presented in Figures I and J of S2 File.

### 4.4 Effect of monsoon on the energy system

Interesting observations were made in the simulations regarding the effect of monsoon on the energy system. Monsoon in India normally starts from June and lasts till September and the starting period depends on a particular region. In India solar is a constant resource for energy. However, in the period of monsoon, there is reduced solar activity due to cloudy and rainy days. But in the monsoon period it was observed that there is a substantial increase in wind resources as shown in Fig 4. For the Western region of India a particular week in the summer months and in the monsoon period shows the flexible operation mode of the RE system, as depicted in Figures A and B of S2 File. In the period of low solar radiation, wind and to some extent hydro balance the system to provide electricity and to keep the energy system running without power failure.

## 5. Discussion

The installation of HVDC transmission grid between the sub-regions enables significant decrease in the cost of electricity and in installed capacities of RE technologies in a 100% RE based system. The benefit due to grid integration varies for different regions of the world [41, 68]. For the SAARC region, benefit due to grid integration seems to be marginal as local storage options seem to be more cost effective than transmission of the electricity. The regional interconnection helps to decrease the cost of storage technologies needed but in a region where there is a high influence of solar power in the system and almost stable solar conditions all around year, batteries are required to store this energy to help balance the night time demand. The solar PV-battery system is a cheaper option than importing electricity via grids.

The total levelised cost of electricity in the SAARC region decreased from 71.6 €/MWh for the region-wide open trade scenario to 69.6 €/MWh for the country-wide open trade scenario and 67.2 €/MWh for the area-wide open trade scenario. The total annualized cost of the system decreased from 187 b€ to 174 b€ from the region-wide to area-wide scenario. The capital expenditure for the system decreased from 1539 b€ to 1421 b€ from the region-wide to the area-wide open trade scenario, respectively. For the country-wide and the area-wide open trade scenario, the cost incurred from installations of HVDC transmission lines is compensated by a decrease in installed capacities of electricity generation sources and storage capacities, which enable lower efficiency losses and import of low cost electricity from other regions. The installation of HVDC lines may not cover the non-electrified people in the rural areas of the SAARC region, which in fact is home to the largest non-electrified population in the world. The best way to bring electricity to these people is to install RE-based mini-grids and solar home systems depending on the population density. Also, grid extension can be a solution for the people living near the grid [69, 70].

PV technologies play a vital role in the SAARC region as it has the highest share of the installed capacities for a 100% RE energy system. The installation of distributed, small-scale PV and more centralized, utility-scale PV has already achieved grid-parity and respective profitability in most parts of the world [57, 71].

The storage requirements for the SAARC region are mainly based on batteries due a high influence of solar PV on the system. The batteries provide 74%–91% of the total stored electricity. The other storage technology which plays a big role in the region-wide scenario is the A-CAES storage which acts as a mid-term storage for storing wind energy and discharging at times of low solar radiation [72]. The impact of A-CAES on the system decreases as the level of grid integration increases due to transferring of electricity via grids over a larger area being more economical than mid-term storage.

The integrated scenario presents a possibility to cover the projected natural gas demand in the industrial sector (except for demand in power generation and residential use) by flexible generation of synthetic natural gas (SNG), and providing clean water in water stressed areas by SWRO desalination. Providing clean water in the SAARC region is most important in the future because lot of regions are under severe water stress. The flexibility provided by integrated scenario to the system is most useful in compensating seasonal fluctuations. The abundance of solar and wind resources in all the sub-regions of SAARC is sufficient to cover additional demand for electricity required for producing 190.7 TWh_LHV_ of SNG and 298.4 billion m^3^ of renewable water. The total electricity required for gas synthesis and SWRO desalination water is 1619.3 TWh_el_ which leads to additional installations of 491 GW of PV and 449 GW of wind energy. The additional demand for gas synthesis leads to a substantial increase of electrolyser units of about 95 GW (+83%) compared to the area-wide scenario. The cost of desalinated water obtained is 1.2 €/m^3^ and the cost of SNG is 141.8 €/MWh.

The different processes used for converting RE sources to electricity give rise to heat as a by-product. The heat generated from biogas and biomass CHP plants, waste-to-energy incinerators, gas turbines, electrolysers and methanation plants can be used for the heating demand in the industrial sector, which has not been integrated into this system. Also, curtailed electricity can be converted to heat and the excess heat can be stored in heat storage and used when required by the heat sector. The area-wide open trade scenario generates usable heat of 595 TWh_th_ per year, for the region-wide scenario it is 533 TWh_th_ per year, and for the integrated scenario it is 745 TWh_th_ per year. The higher usable heat in the integrated scenario is due to a higher curtailment of electricity and more SNG production. The heat generated as a by-product of biomass and biogas plants is evenly distributed over the year. The demand due to the cooling sector is included in the electricity demand; therefore, no additional demand for cooling is considered.

The self-consumption of the generated electricity from PV plays a vital role in the power sector and has a noticeable impact on the system parameters. The comparison between the decentralized system and centralized system gives vital insights into the total annual costs of the system. The total annualized cost for a more centralized 100% RE system is 1.1%, 1.2% and 3.1% lower than decentralized system for region-wide, country-wide and area-wide scenarios, respectively. However, potential positive effects at the distribution grid level and a lower risk level of power cuts has been not taken into account in the modelling. The target function used for prosumers is different than for a centralized generation, and this gives an additional costs to the system. Prosumers tend to reach minimum annual cost of electricity consumption. To get the most out of PV self-consumption, its LCOE must be lower than the grid electricity purchase price but it can be higher than the total system LCOE. In addition to prosumers’ higher electricity generation cost, there is a tendency to increase the cost of the system by installing more flexible options like low cost RE or more storage capacities, which induce a disturbance in the system demand profile. However, the peak demand of the entire system is reduced marginally (Fig 4), particularly the noon time demand is reduced by PV prosumers. PV self-consumption can be particularly valuable in area constrained regions of SAARC, since rooftop area can be utilized for local electricity generation, which in turn reduces losses incurred due to electricity transmission.

There has been no study performed for a 100% RE scenario for the SAARC region so far, connecting the various countries for future electricity trading. Apart from India there are no studies on high shares of renewables for the future for other countries in the SAARC region. However, future scenarios for India seem to be lacking in some aspects. According to Teske et al., [23, 39, 40], renewables would contribute 56–69% and 92–93% for 2030 and 2050, respectively, to the total electricity generated. The installed capacities of the renewables will reach 548–770 GW and 1356–2240 GW by 2030 and 2050, respectively. According to our results, for a 100% RE based system for 2030 the total installed capacity is 1105 GW, of which solar PV contributes 756 GW and onshore wind contributes 204 GW. Abhyankar and Phadke [37] simulated various scenarios for an hourly grid dispatch model for the year 2047. In the minimum emissions scenario, solar will have the highest installed capacity with 930 GW and wind with 472 GW. The results from our simulation for 2030 also emphasize solar playing a major part in the electricity generation mix followed by wind. The excellent solar conditions and rapid cost reduction of solar systems would play a vital role in the future.

In a 100% RE scenario simulated by TERI and WWF-India [9] for the year 2051, the total installed capacity of renewables would be 2870 GW, of which solar PV would contribute 1200 GW, offshore wind 1113 GW and onshore wind 117 GW. According to our results, solar PV contributes much more than wind energy due to excellent solar conditions in India. Wind will play a vital role in periods of monsoon when there is low solar radiation. In the simulation for this research, offshore wind was not included in the study. For the state of Kerala in India, WWF-India and WISE [36], have done a study on a 100% RE scenario for the year 2050. The results from the study indicate an energy system in which PV contributes 51% and wind contributes to 24% to the total electricity generation mix. The results are in accordance with the results of this study for solar PV, which contributes more than 50%. However, the influence of wind on the system is less.

The Powergrid Corporation of India [38], conducted a study of powering the entire country’s electricity demand through the deserts situated in the Western and Northern part of India. All the regions would be connected via HVDC transmission lines. It was assumed, that even in 2050, coal will play a major part (50%) in the generation mix. This is in contradiction with the results obtained from our study, which indicates that a 100% RE based system is possible in 2030. In addition, it is in strong contradiction to the COP21 agreement for a net zero carbon emission target in the world by the middle of the 21^st^ century [22].

The IEA recently published its ‘India Energy Outlook 2015’ [11], which projects installed capacity of 182–221 GW of solar PV and 134–160 GW for other renewables in India for the year 2040, which is in drastic contrast to the findings of this study. The cost assumptions used in the IEA study for India seem to be questionable. The PV LCOE is assumed to be 82 USD/MWh (61.5 €/MWh at 1.33 USD/€), which seems to be too high for the year 2040. For European solar PV power plants, capital expenditures of 850 €/kWp have already been achieved in 2015 and are expected to further decrease to 470 €/kWp in 2030 [71]. According to KPMG [73], the installed capacity for PV will be 166 GW in 2024/25, compared to 2033/34 as predicted by IEA. KMPG assumes 550–670 USD/kWp capex for utility-scale PV plants for the year 2025, which is even lower than assumed in this study. The IEA [11] report does not reflect the already achieved cost reductions of solar PV nor the future cost reduction potential, which is in drastic contrast to the more market related reports [71, 73]. Moreover, the IEA report is also in drastic violation to the set renewables and in particular solar energy targets of the Government of India [31, 74].

The year on year variation in the renewable energy resources is less as compared to daily or monthly variation. Using a different year for solar PV and wind time series would not affect the installed capacities dramatically. As the energy system in the SAARC region is dependent on solar PV, variation in solar radiation is negligible in different years. Also, forecasting errors to the reality are very low. The forecasting errors on a 24 hour time scale are shifting of resource than the amount of resource. These resource fluctuations can be handled by the flexibility options used in the modelling.

There are some limitations of the approach which is used in this study. The use of overnight approach for simulation rather it being a transition. In the real world conditions, a transition of an energy system to a fully sustainable is a better representative. The applied resource limits are dependent on local acceptance. However due to that we have used rather low number of maximum area usable for energy generation, such as a 4% area limit for wind energy, but this would have a rather low impact on agricultural production. The cost of land for installation of solar and wind power plants will be considered in the future, even though they form the smallest component of the total capital expenditure. According to the government of India’s benchmark cost of installing solar power plants, cost of land form the smallest component of the total cost of installing a solar power plant [75]. Wind turbines can be placed well in areas of agricultural production and not dramatically affecting the harvested crops. In addition solar PV is expected to be mainly placed in zero impact areas as suggested by Denholm and Margolis [76] and Szabó et al. [77], such as rooftops, landfills, contaminated industrial and mining sites and further barren land. Power grids are only modelled for interconnecting the sub-regions, but not within the sub-regions, due to lack of respective data and existing modelling constraints.

The results obtained show a low LCOE for the year 2030 in all the scenarios considered in this study. The growth of RE policies in this region has been remarkable in recent years, particular so in India with ambitious projects from the Ministry of New and Renewable Energy [78, 79] and the formation of the International Solar Alliance at COP 21 [34]. These initiatives will support the development of a 100% RE based system for the future in this region. The obtained results from this study can be compared to recent alternatives for non-renewable low carbon technology options in Europe such as nuclear energy, natural gas and coal carbon capture and storage [80], which can partly comply with the climate change mitigation policy for a low carbon based energy system. According to Agora Energiewende [80], the LCOE of the alternatives are 112 €/MWh for a new nuclear plant (assumed for 2023 in the UK and Czech Republic), 112 €/MWh for gas CCS (assumed for 2019 in the UK, and 126 €/MWh for coal CCS (assumed for 2019 in the UK). A report by the European Commission [81], indicates that CCS technology will not be available till the year 2030, and a report by Citigroup questions whether it will ever be profitable at all [82]. The results obtained for a 100% renewable energy based system show the available least cost RE electricity generation options, which would help achieve the goal of net zero GHG emissions set at COP 21 [22]. The results of this paper indicate various scenarios where a 100% RE-based system is possible and lower in cost than the high risk options which have disadvantages related to proliferation risk, nuclear melt down, unsolved nuclear waste disposal, CO_2_ emissions from power plants with CCS technology, health risk due to heavy metal emissions from coal fired power plants and diminishing fossil fuel reserves. Also, nuclear fission has limitations similar to those mentioned above. Also, the associated financial and human research and development resources spent will not solve the energy problems in the world [83]. The criteria for a low cost, fully sustainable energy system are not satisfied by the above mentioned alternative options.

## 6. Conclusion and policy implications

In the recent union budget of 2015–2016, India has set renewable energy targets to install 175 GW by 2022, which is comprised of 100 GW of solar, 60 GW of wind, 10 GW of biomass and 5 GW small hydro capacity [84, 85]. The rapid progress in developing renewable energy in recent years coupled with the above policy goals, demonstrate the seriousness of India’s pledge towards climate change and electricity access to all in a sustainable way. According to Ernst & Young [86], India has been ranked fourth in the world in terms of renewable energy attractiveness. India has taken initiative to launch the most powerful solar alliance ever, which would provide India and the sun-belt countries with an ability to collaborate and disseminate the knowledge on solar technologies [34]. During the annual COP 21 held in Paris, critical decisions were taken and supported by India to limit global warming to below 2 degree Celsius [32]. India is one of the most vulnerable countries to the effects of climate change due to the high population, about 70% of which lives in rural areas and are heavily dependent on natural resources. Increased temperatures, erratic rainfall with droughts and floods, and rising sea levels would have a high impact on the people living in India [12, 13].

Previously, India was negative in its approach and took a corner seat in most international conferences, but in Paris the Prime Minister of India introduced the concept of climate justice and drove home the message of sustainable development [26]. The steps taken by the government of India include discouraging the use of fossil fuels by levying 5.3 €/tonne (1 INR = 0.013 €) green tax on coal, plans to control vehicular pollution, and policies on waste management [26].

The results of this research support the policy goals of the Indian Government, predicting the influence of solar on the energy system. Due to the abundant sunlight received, developing and installing solar energy would be the way forward in achieving sustainable development and energy for all. From the simulation results of this study for the year 2030, India would have 700 GW and 191 GW of installed PV and wind capacity.

To achieve the above goals in the desired timeframe, renewable energy may require financial support from the government in the form of subsidies as received by the fossil fuels [87]. But already according to KPMG [73], solar power is cheaper than imported coal and would be cheaper than domestic coal in 2019 without any subsidies. According to the Indian energy minister, a new coal-fired power plant would produce costlier power than a solar plant [28]. Solar is already a cheaper source to produce electricity than coal and it will require more political support and will of the government than financial support to get it implemented at a faster rate.

The government already has in place various incentives for large scale solar power projects, which can reduce the impact of tariff on the distribution companies. The incentives include a bundling scheme, a viability gap funding scheme and a generation based incentive scheme [88].

For the above targets to be reached, it would be supportive to accelerate local demand for renewable energy by providing preferential feed-in tariffs (FIT) and other incentives such as accelerated depreciation, tax holidays, renewable energy funds, initiatives for international partnerships/collaboration, incentives for new technologies, human resources development, zero import duty on capital equipment and raw materials, excise duty exemption, and low interest rate loans [89].

In order to achieve such a sustainable, RE-based future for the Indian economy, policy recommendations include timely availability of alternative, commercially-viable technological solutions across sectors, rapid scaling-up, together with accelerated strengthening of supporting infrastructure. It further advocates the development of appropriate skill-sets, regulatory and institutional frameworks and adequate manufacturing capacities [9].

In the SAARC region, a 100% RE-based system is achievable and the real policy option. Renewable energy sources can cover the electricity demand for 2030 in sectors such as power, SWRO desalination and synthetic natural gas demand by industry using PtG technology. The proposed energy system configuration can handle the hurdle due to the monsoon season quite effectively. The LCOE obtained was from 67.2–71.6 €/MWh depending on the geographical and sectorial integration. The obtained price range for electricity is lower than for non-renewable energy resources while matching climate change targets. The cost of land for installation of renewable energy sources is not included in this study, however as this cost forms a small component it will not substantially alter the cost of electricity. The heating demand in the industrial and residential sectors may be partly covered by the excess heat generated as a by-product of synthetic natural gas generation and conversion of curtailed electricity to heat. In all the scenarios, solar PV plays a vital role in power generation followed by wind energy in all the regions except Sri Lanka, Afghanistan and Pakistan South, where wind is the least cost energy due to very high FLH. For the scenarios, the storage requirements are mainly based on batteries, which provide 74%– 91% of the total stored electricity. The role of other storage technologies is notable, especially A-CAES, which has a vital role in the region-wide scenario as a mid-term storage between batteries and PtG, particularly in areas of high wind and high seasonal variation. The HVDC transmission grid plays a vital role in the transmission of low cost electricity from Afghanistan and India North to Pakistan North, where trading is more cost competitive than local storage technologies available and also due to high demand of electricity. As the level of grid integration increases, economic benefit due to A-CAES is reduced but other storage technologies such as batteries and PtG are still required. A slight increase of 1%–3% in the total cost of electricity because of PV self-consumption is due to the utilization of solar electricity and in particular respective batteries for self-consumption at a higher cost level. The most important advantage of PV self-consumption is the reduction of noon and afternoon peak hours in the year. In the integrated scenario, seasonal SNG storage is substituted by industrial SNG generation for the electricity sector. The system restricts synthetic natural gas production in the case of energy deficit as a major source of flexibility. A 100% renewable energy system for India and SAARC seems to be highly attractive, in particular due to the fact that it costs less than only the subsidies for a coal-based energy system.

## Supporting information

S1 File Table A: Financial assumptions for energy system components [53, 71, 90, 91, 92, 93, 94]
Table B: Efficiencies and energy to power ratio of storage technologies [90].
Table C: Efficiency assumptions for energy system components for the 2020 and 2030 reference years [63, 90].
Table D: Efficiency assumptions for HVDC transmission [55].
Table E: Regional grid electricity costs [56].
Table F: Average full load hours and LCOE for PV single-axis tracking, PV optimally tilted, CSP and wind power plants in SAARC sub-regions.
Table G: Regional biomass potentials and geothermal energy potentials.
Table H: Regional biomass costs.
Table I: Lower limits of installed capacities in the SAARC sub-regions.
Table J: Upper limits on installable capacities in SAARC sub-regions in units of GW_th_ for CSP and GW_el_ for all other technologies.
Table K: Annual industrial gas demand and water demand for year 2030 in the SAARC sub-regions.
Table L: Total LCOE components in all sub-regions of SAARC.
Table M: Prosumer electricity costs, installed capacities and electricity utilization for SAARC.
Table N: Overview on storage capacities, throughput, full cycles and utilization of A-CAES potential per year for the four scenarios.
Table O: Electricity transmission line parameters for the area-wide scenario for SAARC.
(DOCX)

S2 File Figure A: Hourly generation profile for a representative week in a summer month for India West.
Figure B: Hourly generation profile for a representative week in a monsoon month for India West.
Figure C: Hourly generation profile for a net exporter region, Afghanistan.
Figure D: Hourly generation profile for a net importer region, Pakistan North.
Figure E: Hourly generation profile for Sri Lanka.
Figure F: Electricity generation curves for a whole year for area-wide open trade scenario for the SAARC region.
Figure G: Aggregated yearly state-of-charge for storage technologies, battery (top left), A-CAES (top right), PHS (bottom left), gas storage (bottom right).
Figure H: Profile for interregional electricity trade between regions for area-wide open trade scenario (left) and hydro dam storage (right).
Figure I: Energy flow of the system for the region-wide open trade scenario for 2030.
Figure J: Energy flow of the system for the area-wide open trade scenario for 2030.
(DOCX)
