# Supplementary material for: Electricity system based on 100% renewable energy for India and SAARC
Source: PLoS One. 2017 Jul 19;12(7):e0180611. doi: 10.1371/journal.pone.0180611 (PMC5516989; doi:10.1371/journal.pone.0180611)
Supplement: S1 File — Table A: Financial assumptions for energy system components [53, 71, 90, 91, 92, 93, 94] Table B: Efficiencies and energy to power ratio of storage technologies [90]. Table C: Efficiency assumptions for energy system components for the 2020 and 2030 reference years [63, 90]. Table D: Efficiency assumptions for HVDC transmission [55]. Table E: Regional grid electricity costs [56]. Table F: Average full load hours and LCOE for PV single-axis tracking, PV optimally tilted, CSP and wind power plants in SAARC sub-regions. Table G: Regional biomass potentials and geothermal energy potentials. Table H: Regional biomass costs. Table I: Lower limits of installed capacities in the SAARC sub-regions. Table J: Upper limits on installable capacities in SAARC sub-regions in units of GWth for CSP and GWel for all other technologies. Table K: Annual industrial gas demand and water demand for year 2030 in the SAARC sub-regions. Table L: Total LCOE components in all sub-regions of SAARC. Table M: Prosumer electricity costs, installed capacities and electricity utilization for SAARC. Table N: Overview on storage capacities, throughput, full cycles and utilization of A-CAES potential per year for the four scenarios. Table O: Electricity transmission line parameters for the area-wide scenario for SAARC. (DOCX) [file pone.0180611.s001.docx]

**Supplementary Material – S1 file**

**Electricity system based on 100% renewable energy for India and SAARC**

**Ashish Gulagi*^1^, Piyush Choudhary^2^, Dmitrii Bogdanov^1^ and Christian Breyer^1^**

1. Lappeenranta University of Technology, Skinnarilankatu 34, 53850 Lappeenranta, Finland

2. Indian Institute of Technology (BHU), Varanasi, India.

**E-mail: Ashish.Gulagi@lut.fi, Christian.Breyer@lut.fi**

**Table A: Financial assumptions for energy system components [53, 71, 90, 91, 92, 93, 94]**

| Technology | Capex  [€/kW] | Opex fix  [€/kW] | | Opex var  [€/kWh] | Lifetime  [a] |
| --- | --- | --- | --- | --- | --- |
| PV optimally tilted | 550 | 8 | | 0 | 35 |
| PV single-axis tracking | 620 | 9 | | 0 | 35 |
| PV rooftop | 813 | 12 | | 0 | 35 |
| Wind onshore | 1000 | 20 | | 0 | 25 |
| CSP (solar field) | 528 | 11 | | 0 | 25 |
| Hydro run-of-river | 2560 | 115.2 | | 0.005 | 60 |
| Hydro dam | 1650 | 66 | | 0.003 | 60 |
| Geothermal energy | 4860 | 87 | | 0 | 30 |
| Water electrolysis | 380 | 13 | | 0.0012 | 30 |
| Methanation | 234 | 5 | | 0.0015 | 30 |
| CO_2_ scrubbing | 356 | 14 | | 0.0013 | 30 |
| CCGT | 775 | 19.4 | | 0.001 | 30 |
| OCGT | 475 | 14.25 | | 0.001 | 30 |
| Steam turbine | 600 | 12 | | 0 | 30 |
| Hot heat burner | 100 | 2 | | 0 | 30 |
| Heating rod | 20 | 0.4 | | 0.001 | 30 |
| Biomass CHP | 2500 | 175 | | 0.001 | 30 |
| Biogas CHP | 370 | 14.8 | | 0.001 | 30 |
| Waste incinerator | 5240 | 235.8 | | 0.007 | 20 |
| Biogas digester | 680 | 27.2 | | 0 | 20 |
| Biogas upgrade | 250 | 20 | | 0 | 20 |
|  | Capex  [€/(kW_NTC_*km)] | Opex fix  [€/(kW_NTC_*km)] | Opex var  [€/kWh_NTC_] | | Lifetime [a] |
| HVDC line on ground | 0.612 | 0.0075 | 0 | | 50 |
| HVDC line submarine | 0.992 | 0.0010 | 0 | | 50 |
|  | Capex  [€/kW_NTC_^[[1]](#footnote-1)^] | Opex fix  [€/kW_NTC_] | Opex var  [€/kWh_NTC_] | | Lifetime [a] |
| HVDC converter pair | 180 | 1.8 | 0 | | 50 |
|  | Capex  [€/(m^3^∙a)] | Opex fix  [€/(m^3^ a)] | Opex var  [€/m^3^] | | Lifetime [a] |
| Water desalination | 2.23 | 0.09 | 0 | | 30 |
|  | Capex  [€/(m^3^∙h∙km)] | Opex fix  [€/(m^3^∙h∙km∙a)] | Opex var  [€/m^3^∙h∙km] | | Lifetime [a] |
| Horizontal pumping and pipes | 19.3 | 0.39 | 0 | | 30 |
| Vertical pumping and pipes | 15.5 | 0.31 | 0 | | 30 |

**Table B:** **Efficiencies and energy to power ratio of storage technologies [90].**

| Technology | Efficiency [%] | Energy/Power Ratio [h] | Self-Discharge [%/h] |
| --- | --- | --- | --- |
| Battery | 90 | 6 | 0 |
| PHS | 85 | 8 | 0 |
| A-CAES | 70 | 100 | 0.001 |
| TES | 90 | 8 | 0.002 |
| Gas storage | 100 | 80*24 | 0 |

**Table C:** **Efficiency assumptions for energy system components for the year 2030 [63, 90].**

|  | η_el_ [%] | η_th_ [%] |
| --- | --- | --- |
| CSP (solar field) |  | 51 |
| Steam turbine | 42 |  |
| Hot heat burner |  | 95 |
| Heating rod |  | 99 |
| Water electrolysis |  | 84 |
| Methanation |  | 77 |
| CO_2_ scrubbing |  | 78 |
| CCGT | 58 |  |
| OCGT | 43 |  |
| Geothermal | 24 |  |
| Biomass CHP | 40 | 45 |
| Biogas CHP | 42 | 43 |
| Waste incinerator | 34 |  |
| Biogas upgrade |  | 98 |

**Table D:** **Efficiency assumptions for HVDC transmission [55].**

|  | Power losses |
| --- | --- |
| HVDC line | 1.6 % / 1000 km |
| HVDC converter pair | 1.4% |

**Table E:** **Regional grid electricity costs [56].**

| Region | Electricity costs [€/MWh] | | |
| --- | --- | --- | --- |
|  | Residential | Commercial | Industrial |
| Total area | 83 | 91 | 97 |
| India East | 81 | 92 | 100 |
| India Centraleast | 81 | 92 | 100 |
| India West | 81 | 92 | 100 |
| India Centralwest | 81 | 92 | 100 |
| India North | 81 | 92 | 100 |
| India Northwest | 81 | 92 | 100 |
| India Uttar Pradesh | 81 | 92 | 100 |
| India South | 81 | 92 | 100 |
| India Centralsouth | 81 | 92 | 100 |
| India Northeast | 81 | 92 | 100 |
| Bangladesh | 100 | 78 | 55 |
| Nepal + Bhutan | 110 | 110 | 100 |
| Pakistan South | 55 | 73 | 90 |
| Pakistan North | 55 | 73 | 90 |
| Afghanistan | 150 | 140 | 130 |
| Sri Lanka | 55 | 69 | 82 |

**Table F:** **Average full load hours and LCOE for PV single-axis tracking, PV optimally tilted, solar CSP and wind power plants in SAARC sub-regions.**

| Region |  | Pop.  [mio. Pop] | Electr. demand  [TWh] | PV-single-axis  FLH | PV optimally tilted  FLH | CSP  FLH | Wind  FLH | PV single-axis  LCOE [€/MWh] | PV optimally tilted  LCOE [€/MWh] | | CSP  LCOE  [€/MWh] | | Wind  LCOE  [€/MWh] |
| --- | --- | --- | --- | --- | --- | --- | --- | --- | --- | --- | --- | --- | --- |
| Total area |  | 1922 | 2597 | 2071 | 1645 | 1922 | 1934 | 28 | 31 | 62 | | 61 | |
| India East |  | 159 | 181 | 1870 | 1535 | 1683 | 1263 | 30 | 33 | 70 | | 84 | |
| India Centraleast |  | 154 | 47 | 1995 | 1595 | 1910 | 1608 | 29 | 32 | 61 | | 66 | |
| India West |  | 156 | 313 | 1995 | 1624 | 1860 | 2394 | 29 | 31 | 63 | | 44 | |
| India Centralwest |  | 159 | 303 | 1950 | 1579 | 1785 | 2298 | 29 | 32 | 66 | | 46 | |
| India North |  | 34 | 77 | 2515 | 1878 | 2277 | 2440 | 23 | 27 | 51 | | 43 | |
| India Northwest |  | 161 | 375 | 2011 | 1633 | 1968 | 1626 | 28 | 31 | 60 | | 65 | |
| India Uttar Pradesh |  | 230 | 200 | 2100 | 1639 | 2165 | 1748 | 27 | 31 | 54 | | 61 | |
| India South |  | 166 | 244 | 1946 | 1536 | 1597 | 2174 | 29 | 33 | 73 | | 49 | |
| India Centralsouth |  | 128 | 331 | 1937 | 1559 | 1653 | 2431 | 29 | 32 | 71 | | 44 | |
| India Northeast |  | 44 | 27 | 1808 | 1525 | 1587 | 998 | 31 | 33 | 74 | | 106 | |
| Bangladesh |  | 186 | 162 | 1843 | 1516 | 1681 | 1151 | 31 | 33 | 70 | | 92 | |
| Nepal + Bhutan |  | 34 | 19 | 2334 | 1789 | 2193 | 1579 | 24 | 28 | 53 | | 67 | |
| Pakistan South |  | 85 | 34 | 2236 | 1775 | 2091 | 2333 | 25 | 28 | 56 | | 45 | |
| Pakistan North |  | 160 | 241 | 2187 | 1697 | 2153 | 1522 | 26 | 30 | 54 | | 70 | |
| Afghanistan |  | 44 | 15 | 2340 | 1802 | 2230 | 3449 | 24 | 28 | 53 | | 31 | |
| Sri Lanka |  | 22 | 28 | 1870 | 1535 | 1683 | 1263 | 30 | 33 | 70 | | 84 | |

**Table G:** **Regional biomass potentials (DBFZ, 2009) and geothermal energy potentials**

| Region | Biomass potential [TWh_th_/a] | | | Geothermal Potential |
| --- | --- | --- | --- | --- |
|  | Solid waste | Solid biomass | Biogas sources | [TWh_th_/a] |
| Total area | 69.6 | 874.9 | 145.7 | 198.4 |
| India East | 5.8 | 74.6 | 14.6 | 6.8 |
| India Centraleast | 5.6 | 66.3 | 14.1 | 3.8 |
| India West | 5.6 | 94.4 | 14.3 | 1.1 |
| India Centralwest | 5.8 | 90.2 | 14.6 | 0.0 |
| India North | 1.2 | 38.6 | 3.1 | 0.0 |
| India Northwest | 5.8 | 90.4 | 14.7 | 0.0 |
| India Uttar Pradesh | 8.3 | 97.7 | 21.1 | 0.0 |
| India South | 6.0 | 79.4 | 15.1 | 0.0 |
| India Centralsouth | 4.6 | 72.0 | 11.7 | 0.0 |
| India Northeast | 1.6 | 35.8 | 4.0 | 122.7 |
| Bangladesh | 7.2 | 53.0 | 6.9 | 33.5 |
| Nepal + Bhutan | 1.3 | 13.5 | 1.8 | 29.6 |
| Pakistan South | 3.2 | 21.0 | 2.5 | 0.0 |
| Pakistan North | 6.0 | 39.6 | 5.2 | 0.0 |
| Afghanistan | 0.0 | 0.0 | 0.0 | 0.8 |
| Sri Lanka | 1.6 | 8.3 | 1.2 | 0.0 |

**Table H:** **Regional biomass costs**.

| Region | Biomass costs [€/MWh] | | |
| --- | --- | --- | --- |
|  | Solid waste | Solid biomass | Biogas sources |
| Total area | -9.5 | 8.0 | 6.7 |
| India East | -10.2 | 8.6 | 8.9 |
| India Centraleast | -10.2 | 7.9 | 8.9 |
| India West | -10.2 | 10.4 | 8.9 |
| India Centralwest | -10.2 | 10.0 | 8.9 |
| India North | -10.2 | 13.3 | 8.9 |
| India Northwest | -10.2 | 9.9 | 8.9 |
| India Uttar Pradesh | -10.2 | 7.7 | 8.9 |
| India South | -10.2 | 8.8 | 8.9 |
| India Centralsouth | -10.2 | 9.9 | 8.9 |
| India Northeast | -10.2 | 12.0 | 8.9 |
| Bangladesh | -10.2 | 5.3 | 3.6 |
| Nepal + Bhutan | -10.2 | 5.3 | 5.5 |
| Pakistan South | -10.2 | 5.3 | 3.1 |
| Pakistan North | -10.2 | 5.3 | 3.1 |
| Afghanistan | 0.0 | 0.0 | 0.0 |
| Sri Lanka | -10.2 | 8.2 | 3.0 |

**Table I: Lower limits of installed capacities in the SAARC sub-regions. Data taken from Farfan and Breyer (2016)**.

| Region | Installed capacity [MW] | | | | |
| --- | --- | --- | --- | --- | --- |
|  | Solar PV | Wind | Hydro  RoR and dams | | PHS |
| Total area | 3461.7 | 23883.5 | 44120.7 | | 22032.0 |
| India East | 35.7 | 0.5 | | 3782.8 | 32.0 |
| India Centraleast | 18.0 | 0.0 | 372.0 | | 0.0 |
| India West | 1304.4 | 3339.1 | 4342.1 | | 9600.0 |
| India Centralwest | 289.9 | 3929.7 | 3212.3 | | 2000.0 |
| India North | 6.8 | 0.0 | 5863.6 | | 0.0 |
| India Northwest | 1281.5 | 7935.5 | 2712.5 | | 0.0 |
| India Uttar Pradesh | 36.2 | 0.0 | 533.6 | | 0.0 |
| India South | 74.2 | 5621.3 | 3933.5 | | 3200.0 |
| India Centralsouth | 203.7 | 2809.9 | 6627.7 | | 7200.0 |
| India Northeast | 0.0 | 0.0 | 1316.8 | | 0.0 |
| Bangladesh | 140.0 | 1.0 | 232.0 | | 0.0 |
| Nepal + Bhutan | 6.0 | 0.0 | 2104.0 | | 0.0 |
| Pakistan South | 0.0 | 105.0 | 26.6 | | 0.0 |
| Pakistan North | 45.5 | 49.6 | 7176.3 | | 0.0 |
| Afghanistan | 5.0 | 0.0 | 315.0 | | 0.0 |
| Sri Lanka | 15.0 | 92.0 | 1570.0 | | 0.0 |

**Table J:** **Upper limits on installable capacities in SAARC sub-regions in units of GW_th_ for CSP and GW_el_ for all other technologies.**

| Region | area | | | Limits [GW] | | | | | | | |
| --- | --- | --- | --- | --- | --- | --- | --- | --- | --- | --- | --- |
|  | [1000 km^2^] | Solar  CSP | Solar  PV | | | Wind | Hydro  RoR | Hydro  dams | | PHS | |
| Total area | 5212 | 79749 | 23456 | | 1751 | | 32 | 35 | 44 | |  |
| India East | 252 | 3849 | 1132 | | 85 | | 3 | 3 | 0 | |  |
| India Centraleast | 174 | 2660 | 782 | | 58 | | 0 | 0 | 0 | |  |
| India West | 509 | 7781 | 2289 | | 171 | | 2 | 4 | 19 | |  |
| India Centralwest | 443 | 6776 | 1993 | | 149 | | 2 | 2 | 4 | |  |
| India North | 331 | 5070 | 1491 | | 111 | | 5 | 3 | 0 | |  |
| India Northwest | 438 | 6708 | 1973 | | 147 | | 2 | 2 | 0 | |  |
| India Uttar Pradesh | 241 | 3686 | 1084 | | 81 | | 0 | 0 | 0 | |  |
| India South | 284 | 4349 | 1279 | | 96 | | 3 | 3 | 6 | |  |
| India Centralsouth | 352 | 5386 | 1584 | | 118 | | 5 | 5 | 14 | |  |
| India Northeast | 255 | 3904 | 1148 | | 86 | | 1 | 1 | 0 | |  |
| Bangladesh | 148 | 2258 | 664 | | 50 | | 0 | 0 | 0 | |  |
| Nepal + Bhutan | 186 | 2839 | 835 | | 62 | | 2 | 1 | 0 | |  |
| Pakistan South | 488 | 7469 | 2197 | | 164 | | 0 | 0 | 0 | |  |
| Pakistan North | 394 | 6032 | 1774 | | 132 | | 3 | 8 | 0 | |  |
| Afghanistan | 652 | 9979 | 2935 | | 219 | | 0 | 0 | 0 | |  |
| Sri Lanka | 66 | 1004 | 295 | | 22 | | 1 | 1 | 0 | |  |

**Table K:** **Annual industrial gas demand (IEA, 2013) and water demand (Caldera et al., 2016) for year 2030 in the SAARC sub-regions**

|  | Annual gas demand | | Annual electricity demand for gas synthesis | Annual water desalination demand | Annual electricity demand for water desalination |
| --- | --- | --- | --- | --- | --- |
|  | [Bcm] | [TWh_th_] | [TWh_el_] | [mil. m^3^] | [TWh_el_] |
| Total | 18.0 | 190.7 | 298.7 | 298437 | 1428.6 |
| India East | 0.9 | 9.9 | 10.9 | 597 | 1.9 |
| India Centraleast | 0.9 | 9.5 | 9.8 | 456 | 1.8 |
| India West | 0.9 | 9.7 | 15.8 | 29030 | 119.4 |
| India Centralwest | 0.9 | 9.9 | 13.3 | 2666 | 13.3 |
| India North | 0.2 | 2.1 | 3.3 | 226 | 4.1 |
| India Northwest | 0.9 | 10.0 | 11.9 | 74945 | 333.4 |
| India Uttar Pradesh | 1.3 | 14.3 | 17.2 | 19325 | 91.7 |
| India South | 1.0 | 10.3 | 10.6 | 7419 | 29.7 |
| India Centralsouth | 0.7 | 7.9 | 13.1 | 9242 | 47.8 |
| India Northeast | 0.3 | 2.7 | 2.8 | 0 | 0.0 |
| Bangladesh | 1.7 | 17.6 | 29.5 | 0 | 0.0 |
| Nepal + Bhutan | 0.0 | 0.0 | 0.0 | 0 | 0.0 |
| Pakistan South | 2.8 | 30.1 | 55.8 | 28910 | 119.9 |
| Pakistan North | 5.3 | 56.6 | 104.7 | 115432 | 568.7 |
| Afghanistan | 0.0 | 0.0 | 0.0 | 10190 | 96.9 |
| Sri Lanka | 0.0 | 0.0 | 0.0 | 0 | 0.0 |

**Table L:** **Total LCOE components in all sub-regions of SAARC.**

| Region-wide | LCOE primary | LCOC | LCOS | LCOT | LCOE total |  |
| --- | --- | --- | --- | --- | --- | --- |
|  | [€/MWh] | [€/MWh] | [€/MWh] | [€/MWh] | [€/MWh] |  |
| Area average | 42.3 | 1.5 | 27.8 | 0.0 | 71.6 |  |
| India East | 44.9 | 1.4 | 26.2 | 0.0 | 72.5 |  |
| India Centraleast | 45.8 | 0.1 | 4.3 | 0.0 | 50.3 |  |
| India West | 41.8 | 1.2 | 26.9 | 0.0 | 70.0 |  |
| India Centralwest | 42.6 | 1.1 | 28.9 | 0.0 | 72.5 |  |
| India North | 46.1 | 0.1 | 15.7 | 0.0 | 62.0 |  |
| India Northwest | 42.3 | 1.8 | 32.8 | 0.0 | 76.9 |  |
| India Uttar Pradesh | 41.2 | 2.6 | 26.5 | 0.0 | 70.3 |  |
| India South | 44.2 | 1.6 | 27.7 | 0.0 | 73.4 |  |
| India Centralsouth | 40.8 | 1.1 | 29.5 | 0.0 | 71.4 |  |
| India Northeast | 50.1 | 1.5 | 3.5 | 0.0 | 55.1 |  |
| Bangladesh | 40.9 | 2.7 | 36.9 | 0.0 | 80.4 |  |
| Nepal + Bhutan | 48.3 | 4.4 | 10.1 | 0.0 | 62.9 |  |
| Pakistan South | 43.0 | 0.5 | 18.7 | 0.0 | 62.3 |  |
| Pakistan North | 39.6 | 1.5 | 28.6 | 0.0 | 69.7 |  |
| Afghanistan | 34.6 | 2.8 | 32.3 | 0.0 | 69.7 |  |
| Sri Lanka | 43.6 | 1.4 | 15.8 | 0.0 | 60.9 |  |
| Country-wide | LCOE primary | LCOC | LCOS | LCOT | LCOE total | export (-)/ import (+) |
|  | [€/MWh] | [€/MWh] | [€/MWh] | [€/MWh] | [€/MWh] | [%] |
| Area average | 41.9 | 1.1 | 25.5 | 1.1 | 69.6 | 6.4 |
| India East | 41.8 | 0.2 | 22.7 | 1.4 | 66.2 | 7.8 |
| India Centraleast | 51.2 | 0.1 | 6.0 | 3.9 | 61.3 | 11.9 |
| India West | 43.8 | 1.4 | 22.3 | 1.5 | 68.9 | -5.1 |
| India Centralwest | 41.7 | 0.8 | 26.9 | 0.9 | 70.3 | 3.6 |
| India North | 46.4 | 1.5 | 13.0 | 2.4 | 63.2 | -20.4 |
| India Northwest | 38.2 | 0.6 | 32.5 | 1.5 | 72.7 | 8.4 |
| India Uttar Pradesh | 41.4 | 1.3 | 21.3 | 1.1 | 65.1 | 2.4 |
| India South | 43.9 | 1.2 | 25.2 | 0.8 | 71.1 | 3.5 |
| India Centralsouth | 40.5 | 0.8 | 29.3 | 0.7 | 71.3 | -3.1 |
| India Northeast | 51.3 | 0.1 | 0.6 | 5.2 | 57.2 | -51.0 |
| Bangladesh | 40.9 | 2.7 | 36.9 | 0.0 | 80.4 | 0.0 |
| Nepal + Bhutan | 48.3 | 4.4 | 10.1 | 0.0 | 62.9 | 0.0 |
| Pakistan South | 44.2 | 1.0 | 15.4 | 1.1 | 61.7 | -5.0 |
| Pakistan North | 39.8 | 1.4 | 28.2 | 0.2 | 69.5 | 0.7 |
| Afghanistan | 34.6 | 2.8 | 32.3 | 0.0 | 69.7 | 0.0 |
| Sri Lanka | 43.6 | 1.4 | 15.8 | 0.0 | 60.9 | 0.0 |
| Area-wide | LCOE primary | LCOC | LCOS | LCOT | LCOE total | export (-)/ import (+) |
|  | [€/MWh] | [€/MWh] | [€/MWh] | [€/MWh] | [€/MWh] | [%] |
| Area average | 41.4 | 0.7 | 22.7 | 2.3 | 67.2 | 15.6 |
| India East | 41.8 | 0.3 | 24.1 | 1.1 | 67.3 | 6.1 |
| India Centraleast | 49.1 | 0.2 | 7.9 | 2.8 | 60.0 | 9.9 |
| India West | 43.1 | 1.1 | 22.4 | 0.9 | 67.4 | -2.5 |
| India Centralwest | 41.5 | 0.8 | 27.3 | 0.8 | 70.5 | 3.5 |
| India North | 45.0 | 0.9 | 12.5 | 3.6 | 62.0 | -42.3 |
| India Northwest | 38.1 | 0.2 | 30.7 | 2.0 | 71.1 | 17.9 |
| India Uttar Pradesh | 40.7 | 1.3 | 23.4 | 0.9 | 66.3 | 2.2 |
| India South | 43.9 | 0.5 | 20.9 | 2.0 | 67.3 | 16.8 |
| India Centralsouth | 40.8 | 0.5 | 27.1 | 0.7 | 69.1 | -1.5 |
| India Northeast | 55.9 | 0.5 | 3.1 | 5.0 | 64.6 | -51.0 |
| Bangladesh | 41.9 | 0.4 | 23.5 | 2.4 | 68.1 | 18.9 |
| Nepal + Bhutan | 46.3 | 0.3 | 3.6 | 3.7 | 53.9 | -45.7 |
| Pakistan South | 44.8 | 1.4 | 13.0 | 1.3 | 60.5 | -7.1 |
| Pakistan North | 42.9 | 0.4 | 10.3 | 9.3 | 63.0 | 53.0 |
| Afghanistan | 32.1 | 1.9 | 23.4 | 6.9 | 64.3 | -68.8 |
| Sri Lanka | 34.2 | 1.3 | 24.3 | 5.9 | 65.7 | -45.2 |
| Integrated scenario | LCOE primary | LCOC | LCOS | LCOT | LCOE total | export (-)/ import (+) |
|  | [€/MWh] | [€/MWh] | [€/MWh] | [€/MWh] | [€/MWh] | [%] |
| Area average | 40.8 | 1.4 | 22.6 | 3.1 | 67.9 | 25.4 |
| India East | 45.2 | 0.4 | 23.4 | 1.0 | 70.0 | 6.6 |
| India Centraleast | 56.3 | 0.5 | 6.3 | 1.7 | 64.8 | 5.3 |
| India West | 45.0 | 1.8 | 22.9 | 1.1 | 70.8 | -2.9 |
| India Centralwest | 44.3 | 0.9 | 25.1 | 0.8 | 71.1 | 2.9 |
| India North | 44.3 | 2.3 | 23.2 | 4.5 | 74.3 | -57.6 |
| India Northwest | 37.8 | 0.5 | 34.5 | 2.1 | 74.9 | 22.6 |
| India Uttar Pradesh | 44.1 | 2.1 | 23.6 | 1.0 | 70.9 | 4.0 |
| India South | 46.9 | 0.7 | 19.8 | 1.8 | 69.2 | 18.4 |
| India Centralsouth | 41.6 | 0.8 | 28.5 | 0.6 | 71.4 | -3.0 |
| India Northeast | 60.2 | 0.9 | 3.9 | 3.9 | 68.8 | -49.2 |
| Bangladesh | 40.4 | 0.6 | 19.8 | 1.6 | 62.4 | 17.1 |
| Nepal + Bhutan | 48.2 | 0.9 | 2.8 | 3.1 | 55.1 | -46.6 |
| Pakistan South | 44.2 | 3.1 | 4.9 | 1.4 | 53.6 | -9.0 |
| Pakistan North | 33.5 | 0.7 | 13.2 | 15.0 | 62.3 | 70.0 |
| Afghanistan | 31.5 | 2.2 | 22.7 | 5.2 | 61.6 | -65.4 |
| Sri Lanka | 34.8 | 1.5 | 21.6 | 4.7 | 62.7 | -47.6 |

**Table M:** **Prosumer electricity costs, installed capacities and electricity utilization for SAARC.**

| Prosumers parameters | Residential | Commercial | Industrial |
| --- | --- | --- | --- |
| Electricity price [€/kWh] | 0.080 | 0.089 | 0.096 |
| PV LCOE [€/kWh] | 0.027 | 0.036 | 0.036 |
| Self-consumption PV LCOE [€/kWh] | 0.033 | 0.043 | 0.043 |
| Self-consumption PV and Battery LCOE [€/kWh] | 0.034 | 0.043 | 0.044 |
| Self-consumption LCOE [€/kWh] | 0.034 | 0.043 | 0.043 |
| Benefit [€/kWh] | 0.046 | 0.046 | 0.053 |
| Installed capacities | Residential | Commercial | Industrial |
| PV [GW] | 30 | 36 | 78 |
| Battery storage [GWh] | 1.8 | 0.5 | 1.5 |
| Generation | Residential | Commercial | Industrial |
| PV [TWh] | 49 | 59 | 126 |
| Battery storage [TWh] | 0.6 | 0.2 | 0.5 |
| Excess [TWh] | 8.8 | 8.7 | 20.5 |
| Utilization | Residential | Commercial | Industrial |
| Self-consumption of generated PV electricity [%] | 81.7 | 85.0 | 83.6 |
| Self-coverage market segment [%] | 7.8 | 7.4 | 7.5 |
| Self-coverage operators [%] | 38.8 | 36.8 | 37.3 |

**Table N:** **Overview on storage capacities, throughput, full cycles and utilization of A-CAES potential per year for the four scenarios.**

|  |  | | Region- wide | Country-wide | Area-wide | Integrated |
| --- | --- | --- | --- | --- | --- | --- |
|  | Battery SC | [TWh_el_] | 0.0 | 0.0 | 0.0 | 0.0 |
| Storage capacities | Battery system | [TWh_el_] | 1.4 | 1.5 | 1.4 | 1.7 |
|  | PHS | [TWh_el_] | 0.0 | 0.0 | 0.0 | 0.0 |
|  | A-CAES | [TWh_el_] | 2.5 | 0.6 | 0.0 | 3.2 |
|  | Gas | [TWh_th_] | 100.2 | 85.4 | 79.1 | 208.0 |
|  | Battery SC | [TWh_el_] | 1.2 | 1.2 | 1.2 | 1.2 |
| Throughput of storage technologies | Battery system | [TWh_el_] | 444.8 | 483.9 | 456.9 | 551.3 |
|  | PHS | [TWh_el_] | 11.2 | 11.5 | 11.1 | 12.5 |
|  | A-CAES | [TWh_el_] | 53.4 | 11.8 | 0.1 | 0.0 |
|  | Gas | [TWh_th_] | 180.8 | 145.0 | 125.8 | 81.4 |
|  | Battery SC | [-] | 320.6 | 320.6 | 320.6 | 320.6 |
| Full cycles per year | Battery system | [-] | 320.1 | 318.0 | 315.1 | 327.7 |
|  | PHS | [-] | 255.0 | 259.0 | 251.3 | 283.3 |
|  | A-CAES | [-] | 21.7 | 21.5 | 26.6 | 25.5 |
|  | Gas | [-] | 1.8 | 1.7 | 1.6 | 1.3 |
| Utilization of  A-CAES potential | Storage used | [TWh] | 2.5 | 0.6 | 3.2 | 3.2 |
|  | Storage available | [TWh] | 70210.6 | 70210.6 | 70210.6 | 70210.6 |

**Table O: Electricity transmission line parameters for the area-wide scenario for SAARC.**

| Region 1 | Region 2 | Length  [km] | Capacity  [GW] | Utilization  [%] |
| --- | --- | --- | --- | --- |
| India East | India Centraleast | 508.0 | 3.3 | 67 |
| India East | India Centralwest | 1810.6 | 0.2 | 77 |
| India East | India Centralsouth | 1718.3 | 2.1 | 73 |
| India East | Bangladesh | 264.6 | 1.7 | 62 |
| India Centraleast | India Northwest | 944.1 | 1.5 | 73 |
| India Centraleast | India Uttar Pradesh | 486.5 | 2.1 | 58 |
| India Centraleast | Nepal + Bhutan | 259.4 | 2.1 | 76 |
| India West | India Centralwest | 485.5 | 4.8 | 64 |
| India West | India Northwest | 848.3 | 3.9 | 72 |
| India West | Pakistan South | 669.9 | 1.1 | 70 |
| India Centralwest | India Centralsouth | 924.9 | 2.6 | 75 |
| India North | India Northwest | 711.1 | 13.5 | 69 |
| India North | Pakistan North | 192.8 | 9.7 | 63 |
| India Northwest | India Uttar Pradesh | 461.7 | 3.3 | 63 |
| India South | India Centralsouth | 306.6 | 5.1 | 53 |
| India South | Sri Lanka | 815.5 | 9.1 | 68 |
| India Northeast | Bangladesh | 321.3 | 5.7 | 74 |
| India Northeast | Nepal + Bhutan | 727.1 | 0.6 | 69 |
| Pakistan South | Afghanistan | 1210.5 | 0.5 | 76 |
| Pakistan North | Afghanistan | 391.2 | 23.0 | 70 |

1. NTC – Net Transmission Capacity [↑](#footnote-ref-1)
