# Supplementary material for: Electricity system based on 100% renewable energy for India and SAARC
Source: PLoS One. 2017 Jul 19;12(7):e0180611. doi: 10.1371/journal.pone.0180611 (PMC5516989; doi:10.1371/journal.pone.0180611)
Supplement: S2 File — Figure A: Hourly generation profile for a representative week in a summer month for India West. Figure B: Hourly generation profile for a representative week in a monsoon month for India West. Figure C: Hourly generation profile for a net exporter region, Afghanistan. Figure D: Hourly generation profile for a net importer region, Pakistan North. Figure E: Hourly generation profile for Sri Lanka. Figure F: Electricity generation curves for a whole year for area-wide open trade scenario for the SAARC region. Figure G: Aggregated yearly state-of-charge for storage technologies, battery (top left), A-CAES (top right), PHS (bottom left), gas storage (bottom right). Figure H: Profile for interregional electricity trade between regions for area-wide open trade scenario (left) and hydro dam storage (right). Figure I: Energy flow of the system for the region-wide open trade scenario for 2030. Figure J: Energy flow of the system for the area-wide open trade scenario for 2030. (DOCX) [file pone.0180611.s002.docx]

**Supplementary Material – S2 file**

**Electricity system based on 100% renewable energy for India and SAARC**

**Ashish Gulagi*^1^, Piyush Choudhary^2^, Dmitrii Bogdanov^1^ and Christian Breyer^1^**

1. Lappeenranta University of Technology, Skinnarilankatu 34, 53850 Lappeenranta, Finland

2. Indian Institute of Technology (BHU), Varanasi, India.

**E-mail: Ashish.Gulagi@lut.fi, Christian.Breyer@lut.fi**


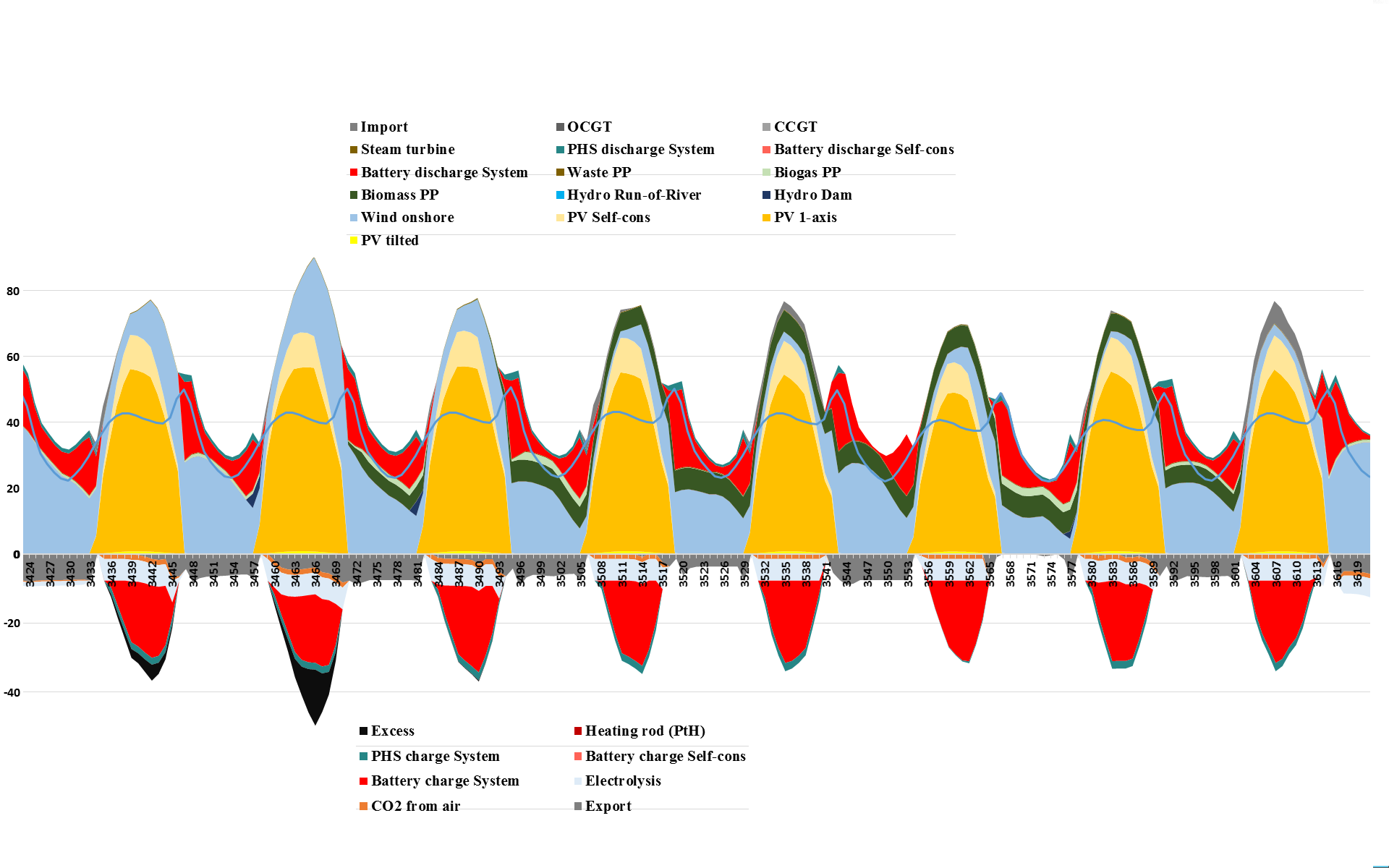


**Figure A:** **Hourly generation profile for a representative week in a summer month for India West.**


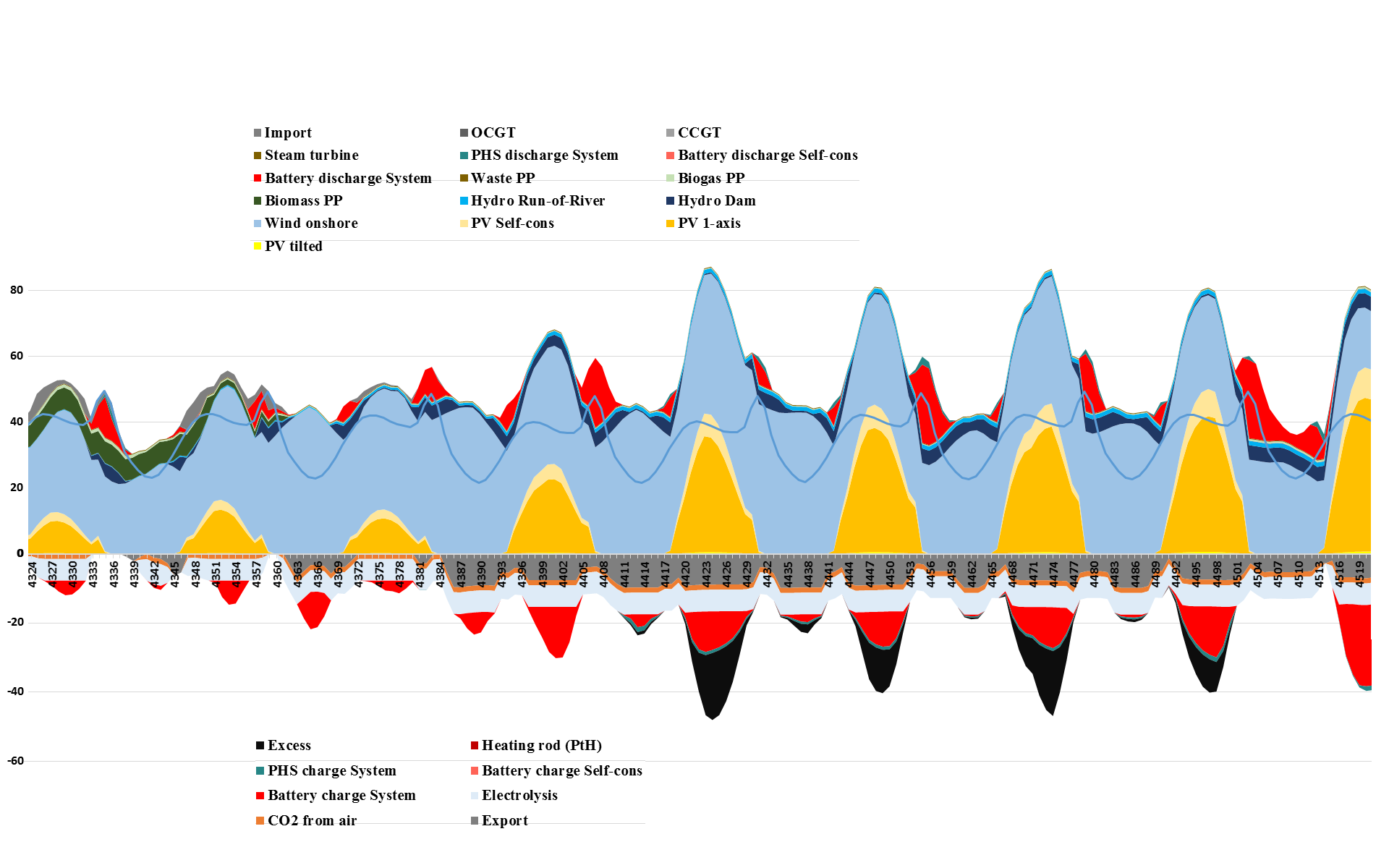


**Figure B:** **Hourly generation profile for a representative week in a monsoon month for India West .**


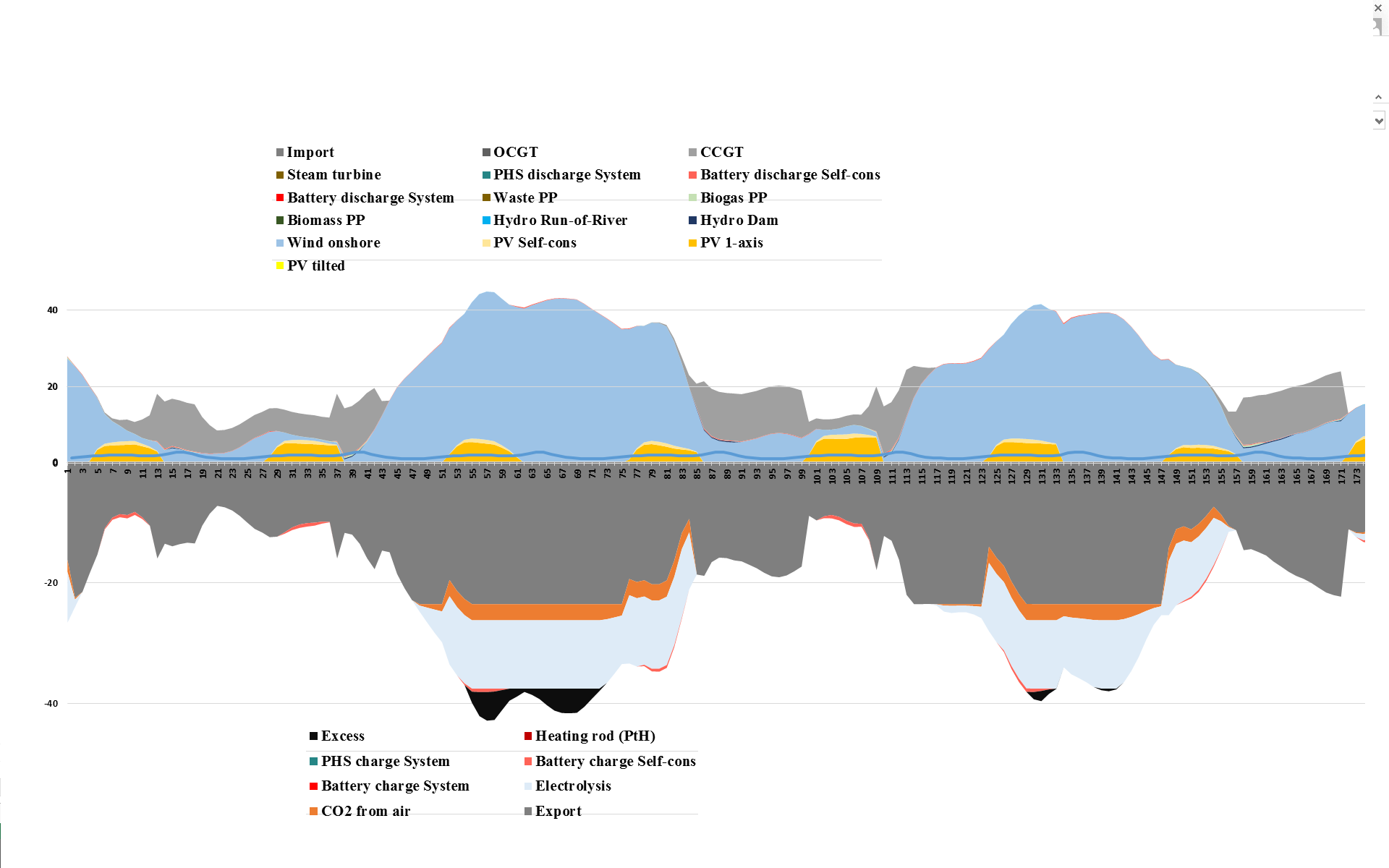


**Figure C: Hourly generation profile for a net exporter region, Afghanistan.**

**
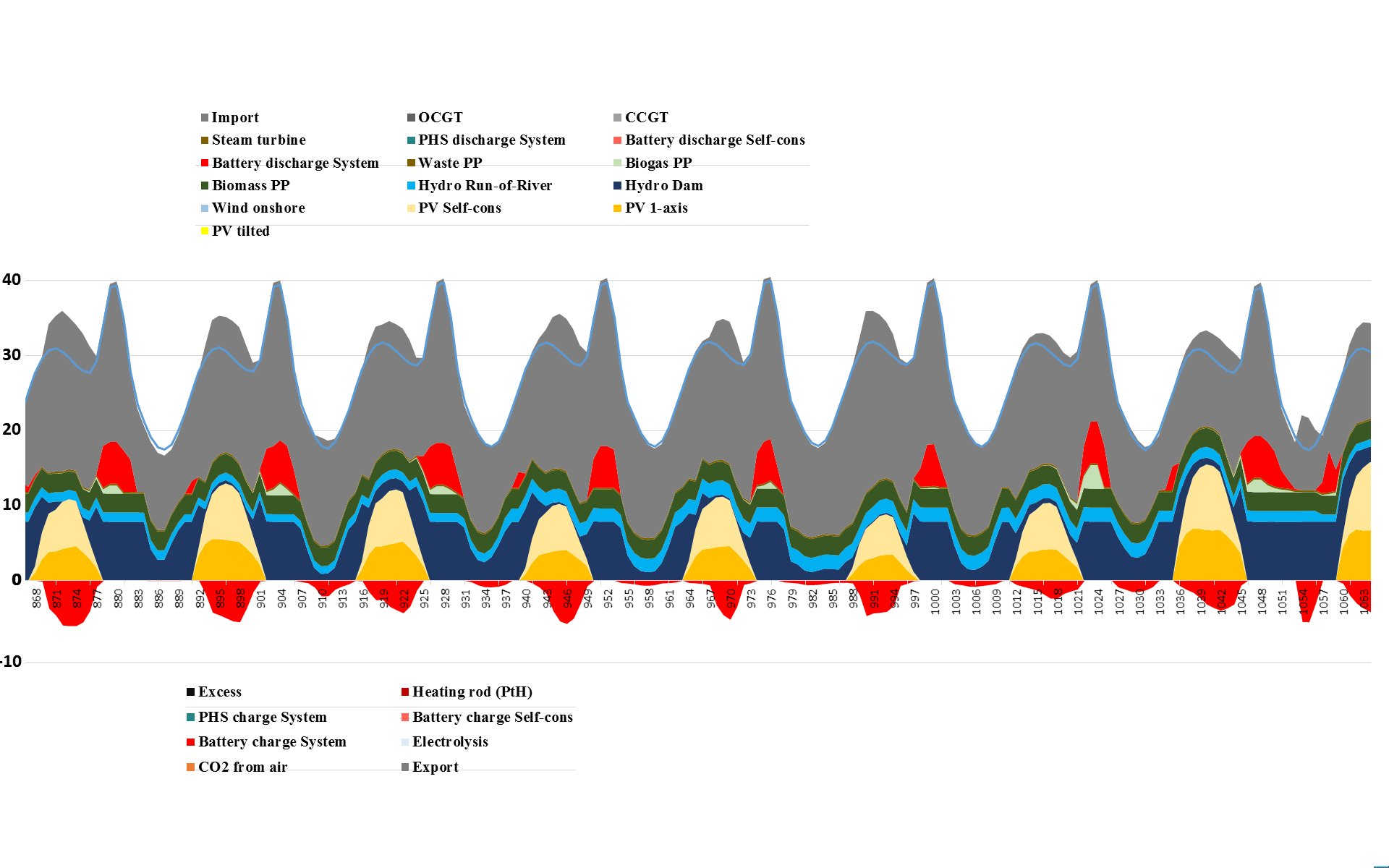
**

**Figure D: Hourly generation profile for a net importer region, Pakistan North.**

**
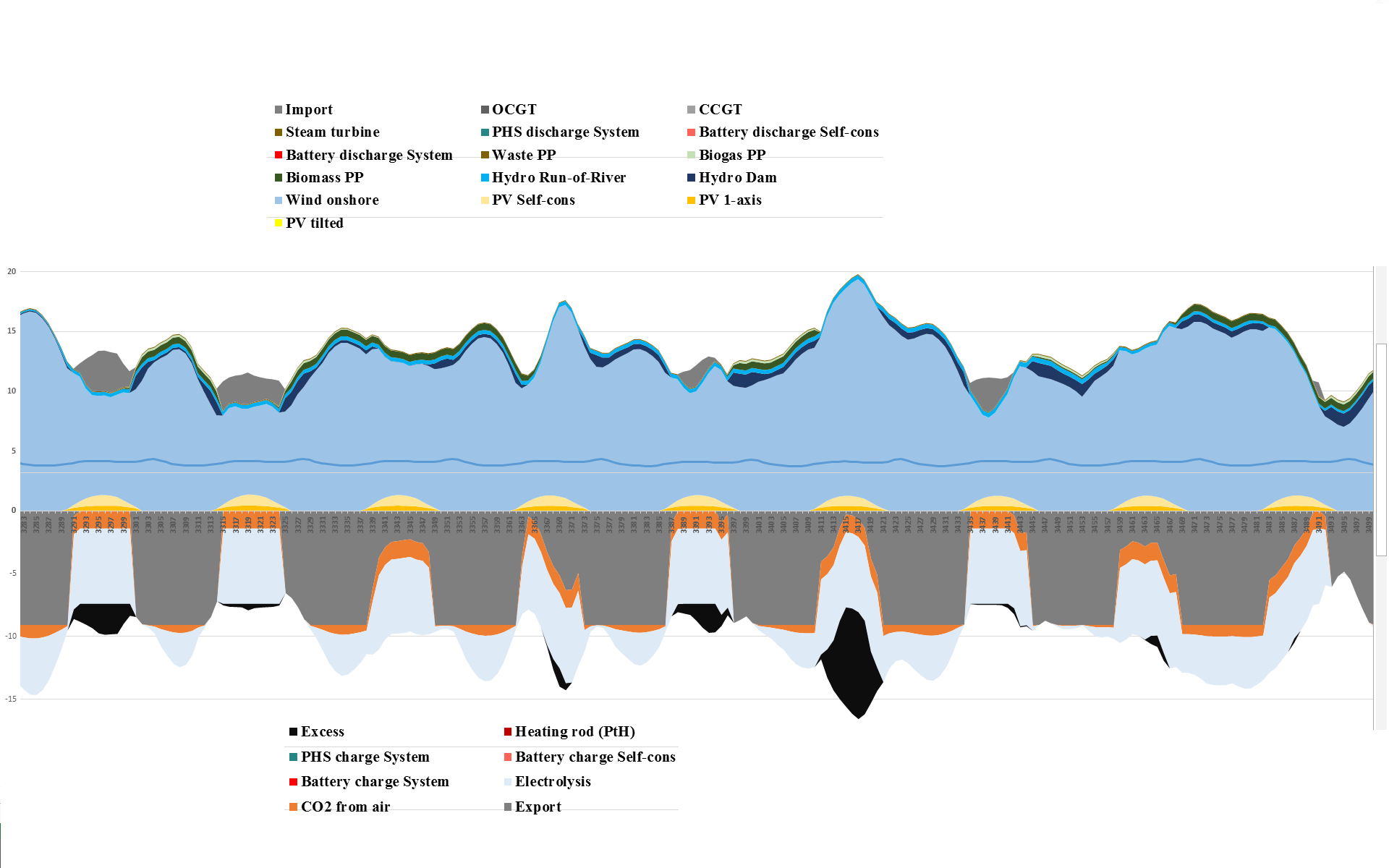
**

**Figure E: Hourly generation profile for Sri Lanka.**


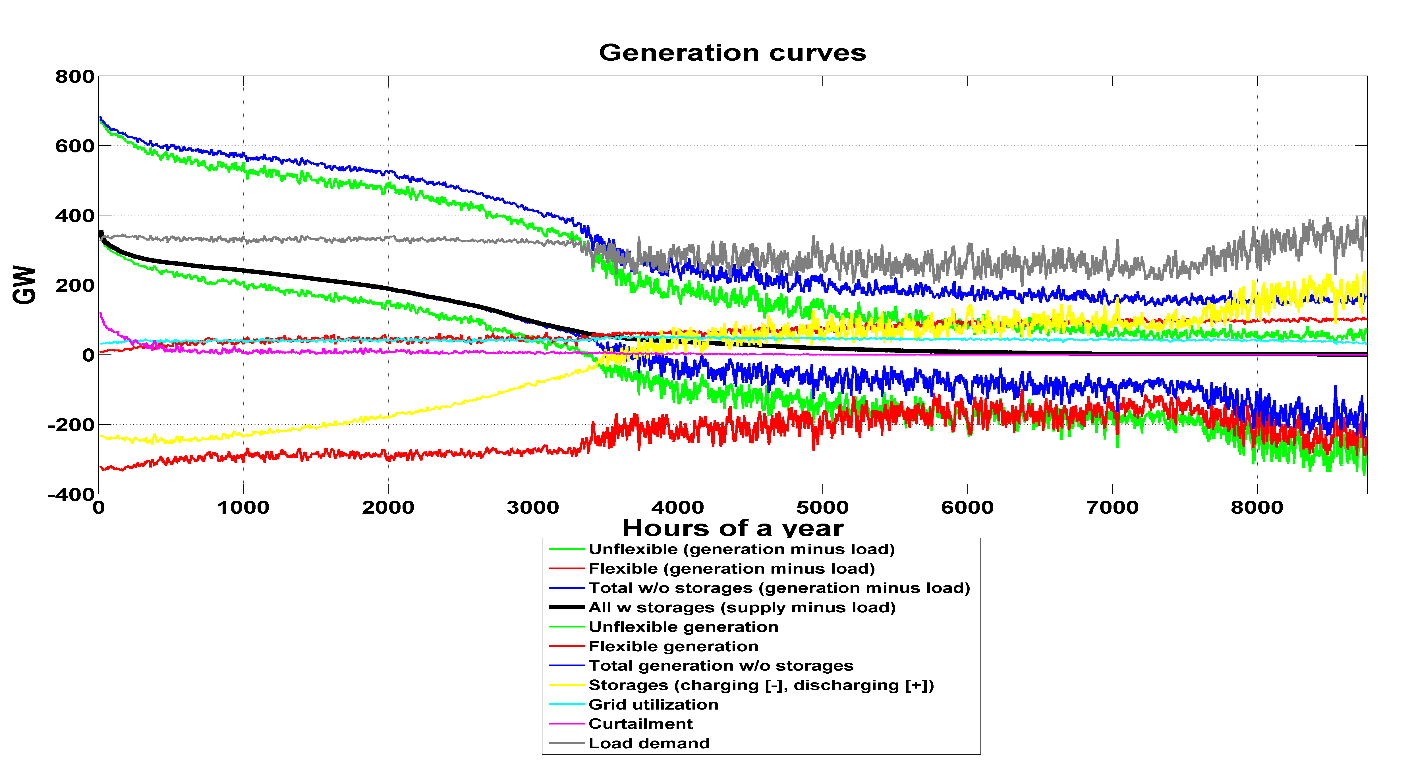


**Figure F:**. **Electricity generation curves for a whole year for area-wide open trade scenario for the SAARC region.**


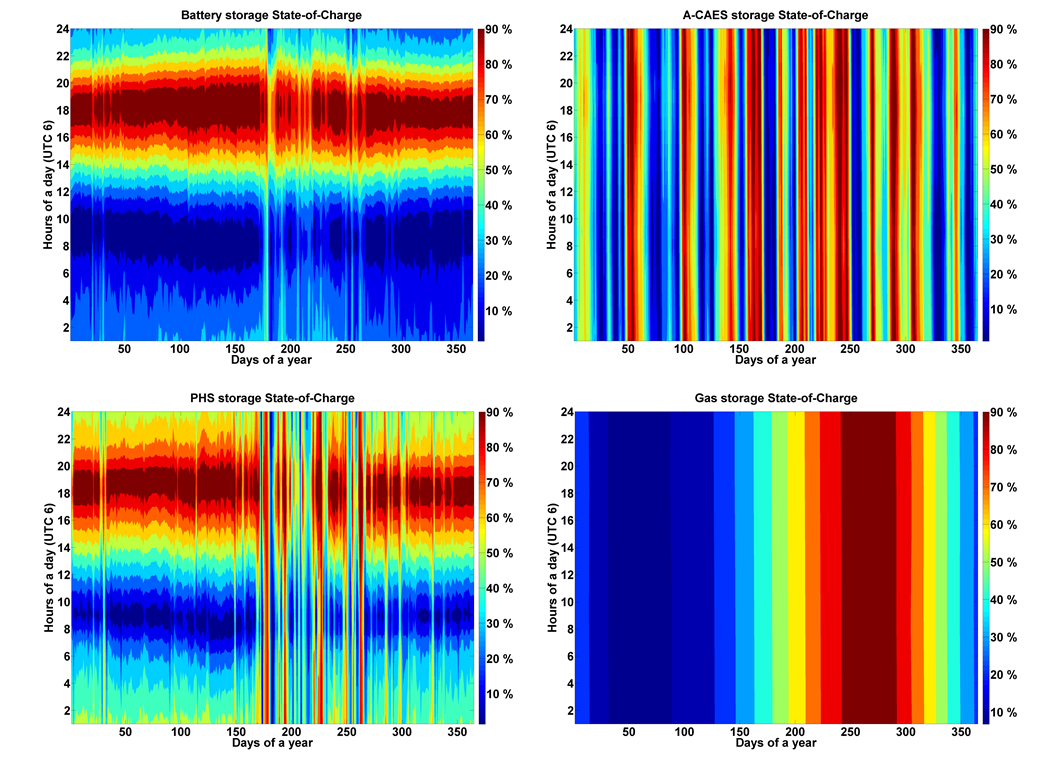


**Figure G:** **Aggregated yearly state-of-charge for storage technologies, battery (top left), A-CAES (top right), PHS (bottom left), gas storage (bottom right).**


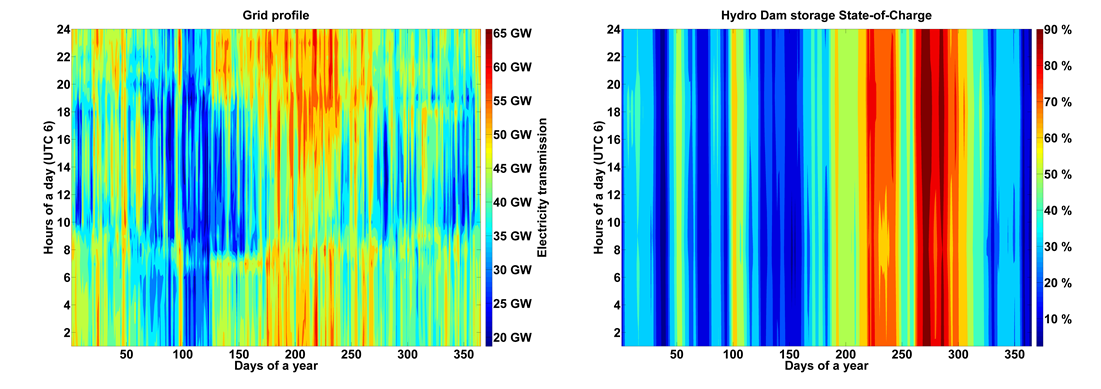


**Figure H:** **Profile for interregional electricity trade between regions for area-wide open trade scenario (left) and hydro dam storage (right**).


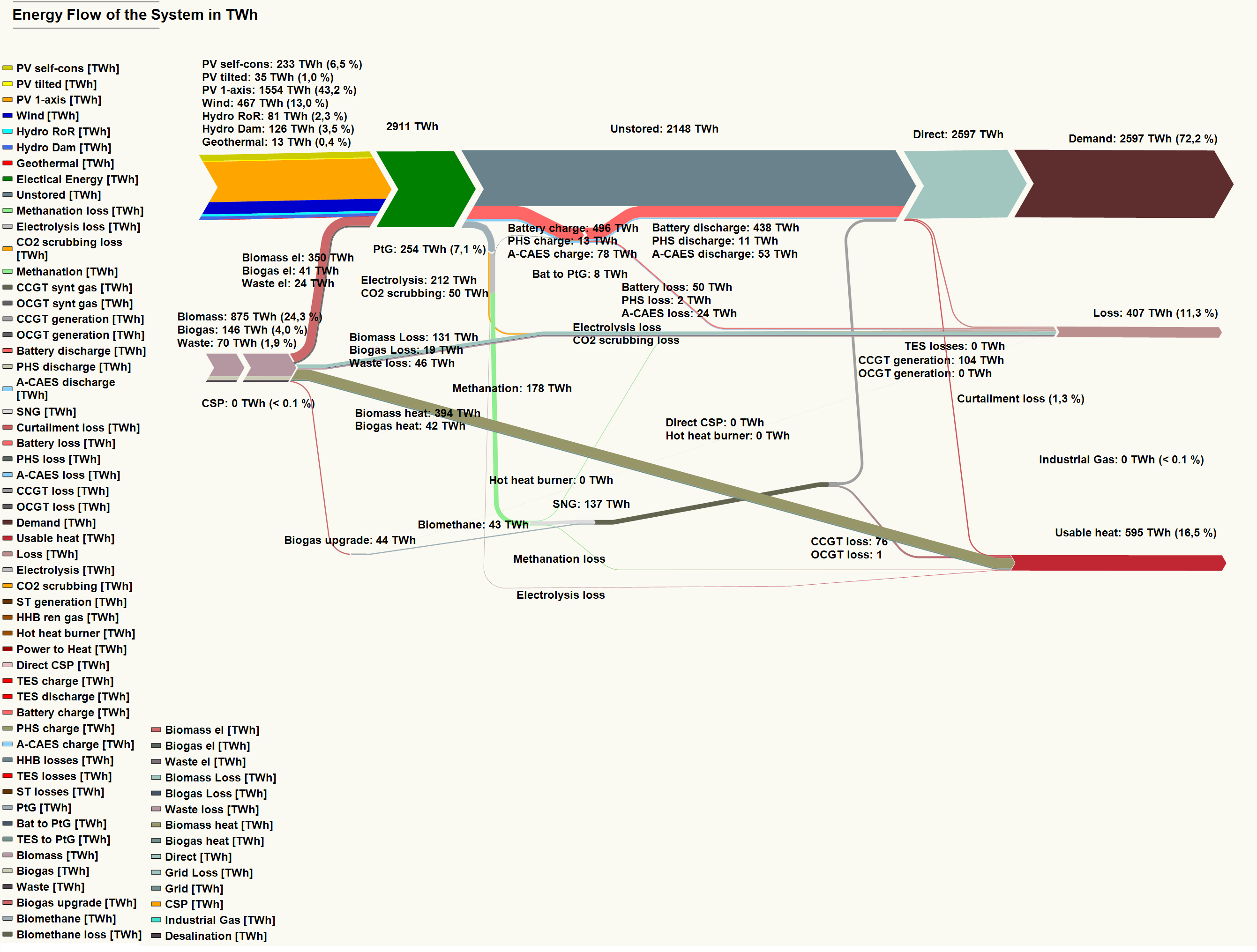


**Figure I:** **Energy flow of the system for the region-wide open trade scenario for 2030.**


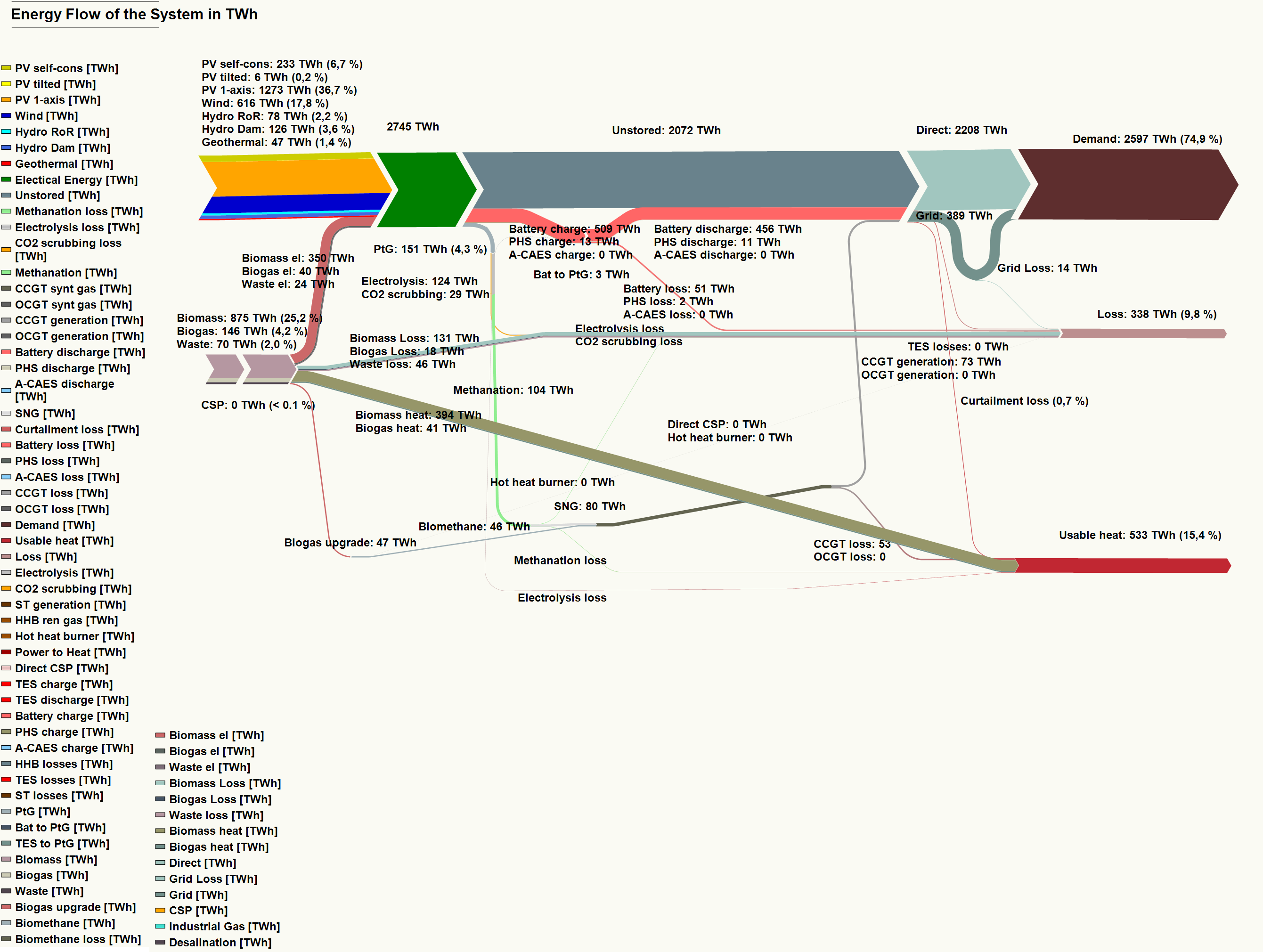


**Figure J:** Energy flow of the system for the area-wide open trade scenario for 2030.
